# Combining Physicochemical and Evolutionary Information for Protein Contact Prediction

**DOI:** 10.1371/journal.pone.0108438

**Published:** 2014-10-22

**Authors:** Michael Schneider, Oliver Brock

**Affiliations:** Robotics and Biology Laboratory, Department of Electrical Engineering and Computer Science, Technische Universität Berlin, Berlin, Germany; University of Michigan, United States of America

## Abstract

We introduce a novel contact prediction method that achieves high prediction accuracy by combining evolutionary and physicochemical information about native contacts. We obtain evolutionary information from multiple-sequence alignments and physicochemical information from predicted *ab initio* protein structures. These structures represent low-energy states in an energy landscape and thus capture the physicochemical information encoded in the energy function. Such low-energy structures are likely to contain native contacts, even if their overall fold is not native. To differentiate native from non-native contacts in those structures, we develop a graph-based representation of the structural context of contacts. We then use this representation to train an support vector machine classifier to identify most likely native contacts in otherwise non-native structures. The resulting contact predictions are highly accurate. As a result of combining two sources of information—evolutionary and physicochemical—we maintain prediction accuracy even when only few sequence homologs are present. We show that the predicted contacts help to improve *ab initio* structure prediction. A web service is available at http://compbio.robotics.tu-berlin.de/epc-map/.

## Introduction

Protein contact prediction identifies potential residue pairs in spatial proximity in the native protein—without knowledge of the native structure itself.

Accurate contact prediction is of great interest and value, as even partial knowledge of residue-residue contacts for a target protein enables the computation of that protein's native structure [1], [2]. Information about native contacts can also be used to guide conformational space search in *ab initio* protein structure prediction [3], [4]. Contact prediction therefore represents an important intermediate step towards the long-standing goal of tertiary structure prediction [5]–[7].

There are five broad categories of contact prediction methods: contact prediction from evolutionary information, from sequence-based machine-learning algorithms, from template structures, from structure prediction decoys and by integrating sequence and structural restraints. They differ in the type of information they use to make predictions.

- *Contact prediction from evolutionary information* leverages the fact that two contacting residues are likely to co-evolve to maintain structural integrity of the protein. Thus, co-evolution signals in multiple-sequence alignments (MSAs) can reveal contacting residues in the protein structure.
- *Machine-learning-based methods* exploit evolutionary sequence information in a slightly different way. They identify common sequence patterns occurring around contacting amino acids. These patterns can be learned and recognized to make contact predictions.
- *Template-based methods* leverage the information contained in structure databases, such as the PDB [8]. They search these databases for appropriate structural templates, using sequence matching or threading and then extract contact information from the retrieved templates.
- *Ab initio protein structure prediction methods* use conformational space search and the *physicochemical information* captured in the energy function to make predictions about contacting residues. These methods generate many low-energy candidate structures and use simple occurrence statistics to identify native contacts.
- *Methods that integrate sequence and structural restraints* use sequence-based predictions and additionally take structural restraints into account. Structural restraints are derived from prior knowledge about protein structures or from templates.

Most of the aforementioned categories of contact prediction methods rely on a single source of information. When no valuable information is available from that source, prediction accuracy deteriorates. This effect is drastic if the number of sequences in the alignment is insufficient or if the correct template cannot be retrieved [9]–[11]. In contact prediction from *ab initio* predicted structures, the quality of information depends on the ability of a search procedure to identify low-energy regions in the energy landscape. If no appropriate regions can be discovered, contact prediction performs poorly.

Methods that use evolutionary information and methods that use physicochemical information are on opposite sides of the spectrum of approaches to contact prediction. Evolutionary information methods are accurate if many sequences are available, but not effective if this information is absent. On the other hand, physicochemical information methods perform well even when only few sequences are available, but do not benefit as much from sequence data as evolutionary methods. Evolutionary and physicochemical information are largely orthogonal information sources. Thus, the combination of those information sources should unlock the synergistic potential of both approaches to perform highly accurate contact prediction.

**Figure 1:**
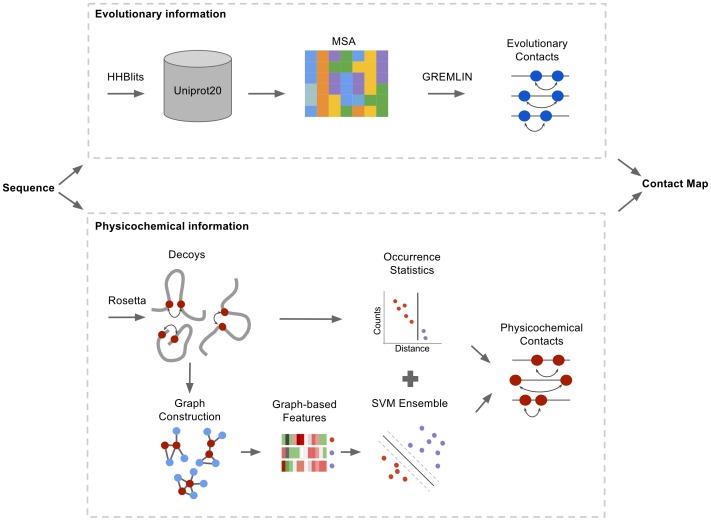
Flowchart overview of EPC-map, combining evolutionary information (upper box) and physicochemical information (lower box). For evolutionary contact prediction, multiple-sequence alignments are constructed by searching the Uniprot20 database with HHblits. GREMLIN is then used to predict contacts from the alignments. For physicochemical contact prediction, decoys are generated with Rosetta. From each decoy, contact graphs are constructed and feature input vectors computed. An SVM ensemble predicts the contact probability from each feature vector. The SVM probability and occurrence statistics predict physicochemical contacts. Lastly, evolutionary and physicochemical contact prediction are combined to form the output of EPC-map.

In this article, we introduce a novel contact prediction method, *EPC-map*, that predicts contacts using two sources of information: evolutionary information from multiple sequence alignments and information from physicochemical energy potentials (EPC-map stands for using Evolutionary and Physicochemical information to predict Contact maps). EPC-map relies on GREMLIN [10], an established method for sequence-based contact prediction, to leverage evolutionary information. To identify and leverage physicochemical information, we present a novel, machine-learning based classifier that uses a graph-based encoding of the structural context of contacts. This classifier distinguishes native from non-native contacts in *ab initio* decoys with unprecedented accuracy. A graphical outline of our method is shown in Figure 1.

In our experiments with 528 proteins, EPC-map reaches 53.2% accuracy on the top scoring 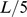 predicted long-range contacts (where 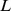 is the length of the protein), increasing the accuracy by 7.8% relative to the state of the art. In our analysis, EPC-map is also the best performing method on proteins from CASP10. Furthermore, we show that EPC-map performs better than contemporary methods, regardless how many sequences are available. We further show that physicochemical information improves prediction in cases where deep alignments are not available, effectively alleviating the main weakness of evolution-based contact prediction. To achieve this, EPC-map does not use any structural information from homologous sequences and is therefore effective when templates are not available. Finally, we show that EPC-map predicted contacts improve *ab initio* tertiary structure prediction.

## Related Work

We review the different approaches to contact prediction following the categorization established above. Of particular interest in our review is the source of information that is leveraged and under which circumstances the methods are applicable.

### Contact prediction from evolutionary information

The earliest methods for contact prediction were based on evolutionary information from multiple sequence-alignments (MSAs). They exploit knowledge of correlated mutations and phylogeny to predict contacts. Over the course of evolution, destabilizing mutations of a specific residue are frequently accompanied by matching mutations of spatially close residues. This is appealing from a theoretical perspective, but initially yielded only poor prediction accuracy [12]. This low accuracy was caused by the presence of transitive correlations obfuscating the information about direct correlations. More recent approaches therefore separate direct and transitive correlations by estimating the inverse covariance matrix of the MSA [13], [14]. This achieves high accuracy but requires on the order of 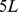 sequences for alignment, where 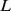 is the length of the protein measured in amino acids [9], [10]. Thus, evolutionary methods are of limited use when only few related sequences are available. For many of these proteins with few sequences, the PDB does not contain any structural homologs [10]; these proteins would therefore benefit most from accurate contact predictions.

### Contact prediction from sequence-based machine learning

Methods in this category employ machine learning to identify sequence patterns indicative of contacts in the protein structure. They vary in the machine learning algorithm they employ and in their training procedure. Researchers have used neural networks [15]–[17], support vector machines [11], [18], hidden-Markov models [19], or random forests [20] to devise prediction algorithms. More recent approaches employ deep learning architectures [21] and deep learning combined with boosting [7]. Improvements in prediction accuracy stem from the application of novel machine learning algorithms, larger training sets, data preprocessing, and/or better training procedures.

Methods in this category are robust when only few sequences are available; they consistently perform well for *ab initio* predictions in the CASP experiments [22], [23]. Nevertheless, these predictors are not routinely used in structure prediction.

### Contact prediction from template structures

A decisively different approach to contact prediction uses information from template structures, making explicit use of structural information available in databases. Methods from this category identify template structures by sequence matching or threading and derive contact predictions from the obtained template [11], [24]. If a good template is found, predictions are highly accurate. This accuracy is further improved through the use of multiple templates [11]. Even though template structures are used to predict contacts, the template retrieval step is essentially based on sequence information, rendering these methods unsuitable for novel folds. The failure to identify a good template then leads to significant loss in prediction accuracy [11].

### Contact prediction from *ab initio* protein structure prediction

Methods based on *ab initio* protein structure prediction sample an energy function to generate many candidate protein structures, called decoys. As native contacts are energetically favorable, they should occur more frequently in these decoys than non-native contacts. It is possible to obtain accurate contact predictions by analyzing the distribution of contacts in those decoys [25]. This approach effectively leverages another source of information than all sequence-dependent methods: The physicochemistry captured in energy functions and encoded in the decoy structures. This approach has been applied successfully to derive consensus distances [26] and energy-weighted occurrences of residue-residue contacts [27]. Related work uses sampling statistics and machine learning to predict native 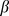-strand contacts [28]. Recently, highly accurate predictions were obtained based on simple occurrence frequencies [25]. Strikingly, this simple heuristic achieved the highest prediction accuracy in an *ab initio* setting at the CASP9 experiment [22] and was among the best in CASP10 [23].

### Contact prediction by integrating sequence and structural restraints

Recent sequence-based methods improve contact prediction by integrating structural restraints into the prediction procedure. Structural restraints have been used as prior probabilities in a pseudo-likelihood approach [10] and by enforcing structural restraints with integer linear programming [29]. These approaches show that the use of structural restraints allows for more accurate contact prediction. Karakas et al. integrate sequence and structure information by coupling sequence-based neural networks with structural templates from fold-recognition [24].

The combination of structure and sequence information is an emerging route towards improved contact prediction. However, combining explicit structure and physicochemical information from structure prediction decoys with evolutionary information has not been attempted yet. As we will show, this approach results in significant performance improvement.

## Methods

### Contact definition and evaluation

Two residues are defined to be in contact if their C*_β_* atoms (C*_α_* for glycine) are within 8 Å in the native structure of a protein. We investigate medium-range and long-range contact predictions. In medium-range (long-range) contacts, the contacting residues are separated by 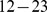 (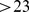) residues in the sequence. For evaluation, we consider the top scoring fraction of 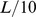, 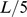 and 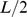 predicted contacts, where 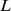 is the length of the protein. Our performance metrics are accuracy (Acc = TP/(TP+FP)) and coverage (Cov = TP*_frac_*/TP *_total_*). Here, TP are true positives: contacts that are predicted and also in contact in the native structure. FP are false positives: contacts that are predicted but not in contact in the native structure. TP*_frac_* are the true positives in the top scoring fraction of the predicted contacts, while TP*_total_* are the total true positives for a protein. Our main analysis focuses on the top scoring long-range contacts as they are most valuable in structure modeling.

### Generation of multiple-sequence alignments

Our method relies on multiple-sequence alignments in two ways. First, the contact graph (defined below) contains information from these alignments, e.g. local sequence conservation of a particular residue. Second, our method incorporates information from sequence-based contact prediction obtained from GREMLIN [10]. These multiple-sequence alignments are generated by searching the query sequence with HHblits [30] (version 2.0.11) against a clustered Uniprot [31] database with maximum pairwise sequence similarity of 20% (dated March 2013).

### Evolutionary contact information

We use GREMLIN [10] to obtain evolutionary information contact scores. We obtained a version of GREMLIN from the authors and run it with default parameters.

### Decoy generation

The generation of protein decoys is the first step towards leveraging physicochemical information for contact prediction. We use the standard *ab initio* protocol in Rosetta version 3.2 to generate decoys [32]. Rosetta performs fragment assembly with a reduced representation of the protein chain, using a knowledge-based force field. Decoys are refined in an all-atom phase, adding side chains and minimizing the decoys' energy in a hybrid physical/knowledge-based all-atom potential.

#### Decoys for training

To generate decoys for training purposes, we use three independent Rosetta runs, each with a different strength of native bias [33]. The goal is to obtain decoys that are far away, relatively close, and very close to the native structure. This will provide us with a good decoy training set, containing a diverse set of positive and negative examples.

The native bias is introduced by three different fragment libraries. We quantify the impact of these biases by using the GDT_TS as a measure of quality of the five lowest-energy decoys for each protein. The GDT_TS measure ranges from 0 if two structures are completely dissimilar to 100 for a perfect structural match. In the first fragment library, we exclude proteins homologous to the target sequence. Decoys obtained using this fragment library are usually far away from the native structure (min/max/mean/median GDT_TS of 10.7/73.5/26.5/24.3). To obtain the second fragment library, we allow fragments from homologous proteins to be included, leading to decoys closer to the native structure (min/max/mean/median GDT_TS of 11.2/99.3/28.1/25.4). We enrich the third fragment library with fragments from the native structure itself, enabling decoys that are even closer to the native structure of the target (min/max/mean/median GDT_TS of 10.4/99.8/37.0/31.2). Note that this use of the native bias is acceptable, as we are only generating decoys for training purposes. For each type of fragment library, we generate 200 decoys, resulting in 600 decoys per protein. From each of these sets of 200 decoys, we retain the 3% with lowest energy.

#### Decoys for prediction

To perform contact prediction, we generate 1000 decoys without homologous fragments, i.e. using the first fragment library, and retain the top 2% based on energy. Thus, our approach does not use any structural information from homologous sequences such as templates. Therefore, we set EPC-map apart from methods that use threading to find templates and extract contacts from them.

### Contact graphs for feature generation

Past research has demonstrated the effectiveness of decoy-based contact predictions using simple occurrence statistics [25]. However, these statistics were gathered on entire decoys, selected by their energy. The energy criterion favors all occurring contacts equally, even the non-native ones, making it difficult to differentiate between native and non-native contacts.

Our approach is based on the insight that the discrimination of native and non-native contacts in decoys must improve significantly if information from the decoy's energy is complemented with information specific to the individual contacts. Our main assumption is that this information is captured by the immediate structural environment of a contact. Thus, we would like to characterize this local environment and learn how to differentiate native from non-native environments.

**Figure 2:**
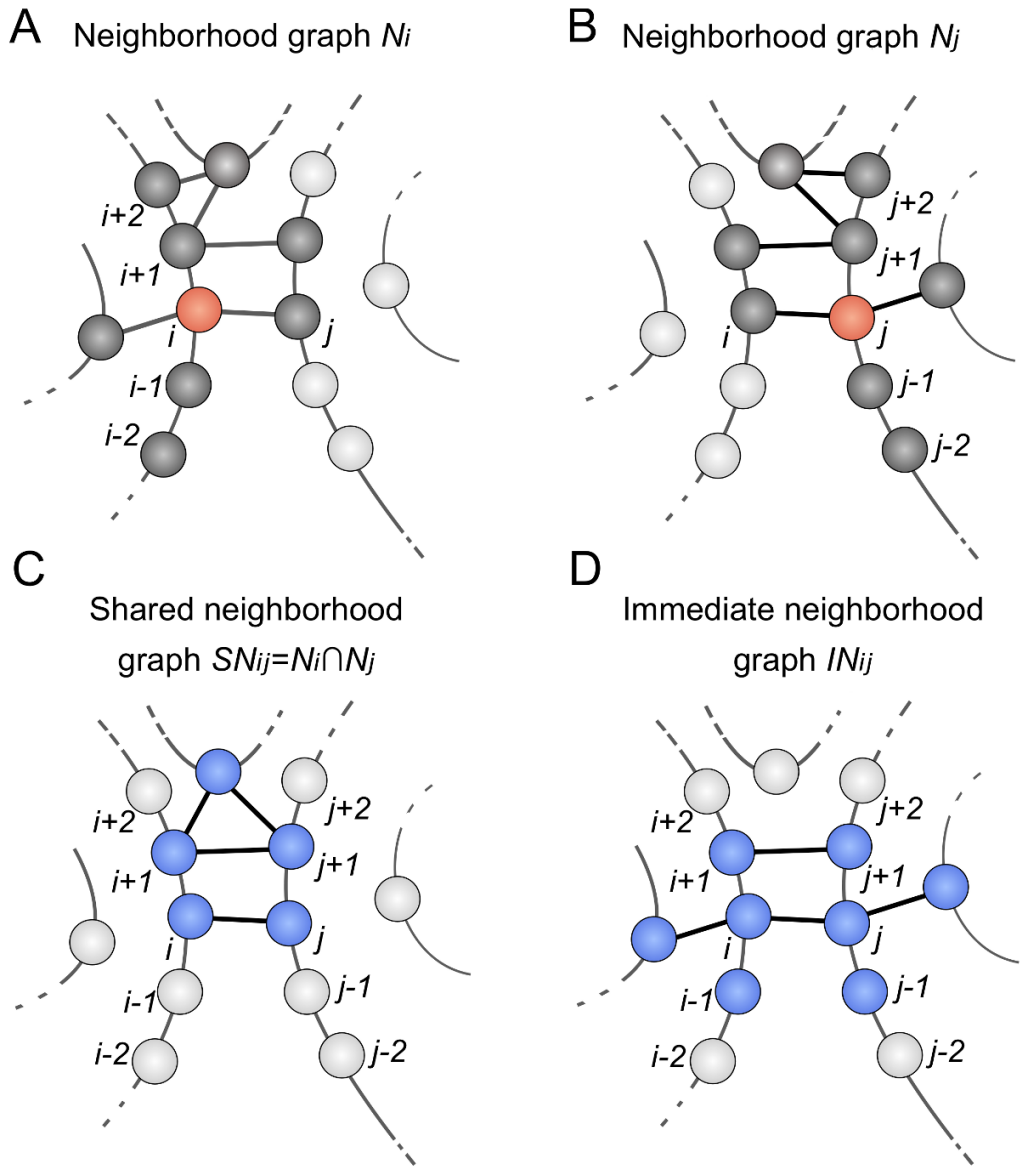
Definition of graphs used to model the neighborhood of the contacting residues *i* and *j*: Nodes represent residues (circles), edges represent contacts (solid black lines). **A:** The neighborhood graph 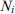 for residue 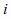 contains all residues in contact with residues 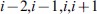, and 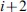 (dark grey). **B:** The neighborhood graph 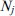. **C:** The shared neighborhood graph 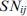 for the contact between residues 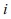 and 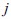 is defined by the intersection of 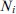 and 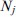. Residues that belong to 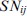 are shown in blue. Shared neighborhood graphs capture the local context of the shared neighborhood of the contacting residues. **D:** The immediate neighborhood graph 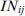 is defined by all residues that are in contact to 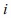 or 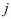. Residues that belong to 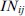 are shown in blue. Immediate neighborhood graphs capture the direct neighborhood of the contacting residues.

To characterize the properties of a contact's neighborhood, we use undirected graphs (refer to Figure 2 for the remainder of this section). In these graphs, nodes correspond to residues and edges connect contacting residues. Nodes and edges are labeled with physicochemical, structural and evolutionary characteristics; these labels are described in the supporting information (Text S1 and Tables S1–S2 in File S1). First, we consider the neighborhood of individual residues. The neighborhood of residue 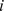 is defined as all residues up to two positions away in sequence, i.e. residues 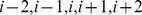, as well as all residues in contact with those, according to the definition of a contact given in Methods. For *α*-helices, the 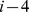, 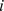, 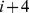 residues are used instead to include the residues with the same facing towards the contact on subsequent helix turns. We capture this notion of neighborhood of residue 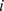 in a neighborhood graph 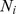 (Figures 2A and B).

To capture the local context of a contact 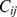 between residue 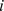 and 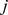, we use two different kind of graphs. The shared neighborhood graph (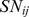) captures the shared neighborhood of the residues 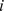 and 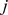 in the context of their sequential neighbors 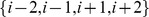 and 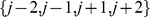, thus being the intersection of 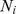 and 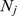: 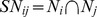 (Figure 2C). Additionally, we capture the local context that directly influences the residues 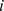 and 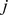 by the immediate neighborhood graph (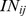) that is formed by all residues in immediate contact to residues 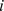 and 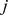 (Figure 2D).

These graphs are the fundamental data structure of our method. We then apply machine learning to learn to discriminate native from non-native graphs (i.e. contacts). The success of learning critically depends on the expressiveness of the employed features, which we will describe next.

### Overview of used features

To capture contact characteristics in decoys, we define 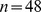 features, each representing a measurable property of residues in contact. Each feature consists of one or several binary or real-valued inputs. All of these inputs are joined into a single vector, which serves as input to a support vector machine (SVM) during training and testing. Note that features defined on graphs are evaluated for the shared neighborhood graphs and immediate neighborhood graphs separately. Thus, each graph feature is present two times in the final input vector which has a length of 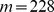.

Features are categorized into seven groups: pairwise, graph topology, graph spectrum, single node, node label statistics, edge label statistics, and whole protein features. We will briefly motivate each of these groups (see also Table 1).

**Table 1:** Overview of the features used for contact prediction. A detailed description of the features is given in the supporting information.

Group	Feature examples	Number of inputs
Pairwise	Chemical type, secondary structure, solvent accessibility, sequence separation, hydrogen bonding, sequence separation from N/C-terminus, contact potential, distance, average distance in ensemble, mutual information	49
Graph topology^a^	Number of nodes, number of edges, average degree centrality, average closeness centrality, average betweenness centrality, graph radius, graph diameter, average eccentricity, number of end points, average clustering coefficient	10
Graph spectrum^a^	Largest two eigenvalues, number of different eigenvalues, sum of eigenvalues, energy of adjacency matrix	5
Single node^a^	Degree, closeness centrality, betweenness centrality, sequence conservation and sequence neighborhood conservation for *i* and *j*	10
Node label statistics^a^	Chemical type of residues, secondary structure descriptors, solvent accessibility, hydrogen bonding, average free solvation energy, 4-bin solvation energy distribution, entropy of labels, neighborhood impurity degree, average distance from centroid, sequence conservation, sequence neighborhood conservation	43
Edge label statistics^a^	Link impurity, 5-bin mutual information distribution, cumulative mutual information, 3-bin contact potential distribution	12
Whole protein	Amino acid composition, secondary structure composition, length class	29

a Graph-based features.

Pairwise features capture properties of the amino acids 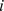 and 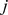, such as the chemical type, secondary structure, and solvent accessibility.

We use topological and spectral graph features to characterize the underlying contact network. For example, nodes in well-packed regions of a decoy will tend to have a higher degree than those in poorly-packed regions. Consequently, contacts in well-packed regions have a higher likelihood of being native. This can be measured by the average degree centrality of the graph.

Node and edge label statistics extract additional information from contact networks to complement topological considerations. For example, native contacts in the protein core should be embedded in a network of hydrophobic residues. This property is captured by the distribution of the chemical nature of neighboring nodes.

Whole protein features specify information about the protein at hand, such as amino acid composition, secondary structure composition and protein chain length.

The individual features from each group are listed in Tables S3-S9 in File S1. A detailed description of each feature and its implementation is also provided in the supporting information (Text S2 in File S1).

#### Software used for feature generation

Several of our features are based on external software. Solvent accessibility and free solvation energies are computed with POPS [34]. We use STRIDE [35] to obtain secondary structure and hydrogen bonding assignments from decoys. Sequence conservation features are computed as described by Fischer et al. [36]. Some of our pairwise features are inspired by Cheng et al. [18] and we use a contact potential introduced in [20]. We construct graphs by using the NetworkX Python package [37] and use the SVM library of scikit-learn [38]. Finally, many of our topological and spectrum features have been shown to be effective for graph classification [39].

Next, we describe how we use these features to train a support vector machine with physicochemical contact information.

### SVM training with physicochemical contact information

A challenging aspect in using support vector machines for contact prediction is that the contact prediction learning problem is inherently imbalanced i.e. there are many more non-native than native contacts in the decoys of our training set (see respective section for details on training set construction). Random undersampling is a common technique to cope with the unbalanced learning problem [40]. However, performing random undersampling leads to information loss because many training instances are not used for learning. Furthermore, the resulting learner might be biased towards the specific training sample, leading to high variance. We reduced this effect by training an SVM ensemble, with each SVM performing its own random undersampling. First, proteins in the training set are randomly split into five non-overlapping subsets. Second, a SVM classifier is trained for each subset with random undersampling by selecting 50 native and 150 non-native contacts. This procedure handles the imbalanced training problem by random undersampling and reduces the effect from information loss and variance by using multiple SVM instances. This yields an SVM ensemble of five different SVM classifiers, one for each subset. Each subset uses approximately 30.000 training instances. Each input in the input vector is normalized by subtracting the mean and dividing by standard deviation.

We use a binning procedure [41] to obtain calibrated probability estimates. The raw SVM output values between the 5 and 95 percentile are grouped into ten bins. Then, the probability of a native contact is computed separately for each bin. We find that this procedure improves prediction accuracy compared to Platt's method [42].

We use the Gaussian kernel for training and determine the cost and the kernel parameter 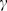 by 10-fold cross-validation on the EPC-map_train data set, optimizing the long-range 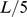 accuracy. Furthermore, probability estimates by binning are obtained by 5-fold cross-validation on the training set. We find that 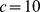 for the soft margin parameter and the Gaussian kernel parameter 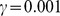 yield the best performance.

### SVM prediction of contacts from physicochemical information

To perform contact prediction for a protein, we consider the 2% lowest-energy decoys generated by Rosetta, using a homology-free fragment library. Each contact present in each decoy is scored by the SVM ensemble. The probability 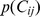 for an individual contact 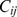 in one decoy is given by:
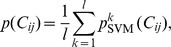
where 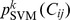 is the probability output value of the 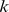-th SVM.

Note that the same contact may appear in multiple decoys. The final score of a contact is the average score over all decoys in the *decoy ensemble* containing that contact:
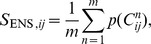
where 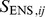 is the score of the contact between residues 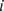 and 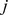, 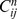 is the contact in the 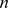-th decoy, 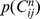 is the output of the SVM ensemble for the contact 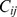 in the *n*-th decoy, and 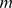 is the number of decoys containing the contact.

### Combination of evolutionary and physicochemical information for contact prediction

Finally, we combine evolutionary and physicochemical information to predict contacts. The output of the SVM system is combined with the frequency 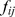 of contact 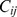 occurring in the ensemble and the score output value of GREMLIN 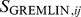:
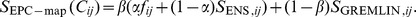



The 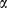 and 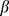 parameters are found by optimizing the 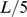 accuracy of long-range contacts by five-fold cross-validation on the training set. Output values from GREMLIN scale differently, depending on how many sequences are available. Furthermore, the performance of GREMLIN is highly dependent on the number of available sequences. Predicted contacts from GREMLIN perform well in template discrimination tasks if 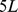 or more sequences are available [10]. This indicates that GREMLIN is accurate for proteins with more than 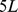 sequences, but does not consistently perform well if less sequences are found. Thus, separate 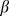 parameters are tuned for proteins with 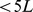 sequences and for proteins with 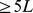 sequences. With this procedure, we supplement GREMLIN's predictions that are already accurate (when many sequences are available) and compensate for GREMLIN's loss in accuracy when only few sequences are available. Final parameters are: 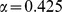 and 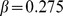 for proteins with 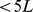 and 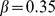 for proteins with 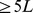 sequences, respectively. The output of our algorithm is the list of contacts in rank order based on their score.

### Data sets

We compiled a non-redundant training set, EPC-map_train, to provide patterns of native and non-native contacts for learning. In addition, we used six test sets (EPC-map_test, D329, SVMCON_test, CASP9-10_hard, CASP10 and CASP10_hard) to evaluate the performance of our method.

#### EPC-map_train

The training set consists of protein chains culled from the PDB using PISCES [43]. The resulting set contains chains of 50–150 amino acids with at most 25% sequence identity and 0–2 Å resolution for X-ray structures. We limited ourselves to smaller chains for training to facilitate rapid method development and testing. From this set we removed: *a)* chains containing chain breaks (a chain break is defined as a distance larger than 4.2 Å between C*α* atoms of two residues adjacent in sequence [20]) and *b)* chains with extended structures and chains whose structure is significantly determined through packing to other chains in their PDB structure or interior bound ligands. To avoid structural redundancy, we performed pairwise structural alignment with Deepalign [44] and removed chains that had a GDT_TS of 60 or more to any other chain in the training set (if the aligned region comprised more than 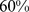 of the smaller protein).

Finally, we removed all chains from the training set that had more than 25% sequence identity or a GDT_TS of 60 or more to any of the chains in the EPC-map_test, D329, SVMCON_test and CASP9-10_hard test sets. From the remaining chains, we removed 15% randomly to form the test set EPC-map_test. The final training set consists of 742 chains. All of our predictions on the CASP10/CASP10_hard data set are performed with a version of EPC-map that uses the 727 training proteins dated before CASP10 (May 2012).

#### EPC-map_test

This test set contains 132 chains randomly selected from the training set as described above. The proteins in this set were not used for training.

#### D329

The D329 data set [20] consists of 329 chains of varying sizes (55–458 amino acids).

#### SVMCON_test

The SVMCON_test data set is comprised of 48 medium-sized protein chains (46–198 amino acids) [18]. We excluded one protein (1aaoA), because it is listed as a theoretical model in the PDB.

#### CASP9-10_hard

We used 16 protein chains from the CASP9 experiment and four protein chains from the CASP10 experiment (20 total). Chains in this set contain *only* free modeling domains (FM category in CASP) or difficult template-based modeling (TBM/FM category) domains. Note that proteins containing at least one FM or TBM/FM domain are also excluded from this set. These proteins are among the most difficult modeling targets, because they do not have many sequence homologs, templates or have unusual folds. Since there is no template information available for these proteins, they represent cases for which contact prediction might be most useful.

#### CASP10

We used 104 proteins for which crystal structures are available from the CASP website at the time of this study.

#### CASP10_hard

We also evaluated our approach on a subset of the CASP10 data set by taking difficult proteins from CASP10. Unfortunately, only four proteins are available from CASP10 that are exclusively comprised by FM or TBM/FM domains. Thus, we selected all CASP10 proteins that contain at least one FM or TBM/FM domain for this evaluation. This results in 14 protein chains.

Importantly, the CASP10 and CASP10_hard data sets allow us to compare our results to all groups that participated in CASP10. The results from the CASP10 methods are available from the CASP website. All of our predictions on the CASP10/CASP10_hard data set are performed with a version of EPC-map that only uses databases and proteins that are dated before CASP10 (May 2012). This allows us to make a fair comparison of EPC-map with all other methods that only had information available that is dated May 2012 or earlier.

### Modeling of contact restraints in Rosetta

In addition to the accuracy of contact prediction, we also quantify the benefits gained from the predicted contacts in *ab initio* protein structure prediction. We use contacts as distance restraints in *ab initio* Rosetta calculations. In other words, we include in Rosetta's energy function the degree to which predicted contacts are present in a decoy. However, contact predictions are likely to contain false positives. Therefore, we do not penalize the violation of a particular predicted contact. Instead, we devise an energy term to maximize the number of satisfied contacts for a given conformation. This is accomplished by incorporating a modified Lorentz function 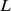 into the energy function of Rosetta:
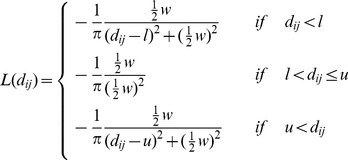
where 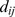 is the distance between residues 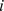 and 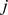 in the decoy. Further parameters of the function are the lower bound 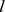, the upper bound 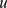 and the half width 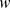. We use 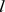 = 1.5 Å, 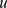 = 8 Å and 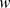 = 1.0. The 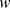 parameter regulates how quickly the energy bonus decreases when 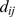 is not within the lower/upper bounds, with 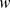 being the half-width i.e., the violation in where the 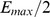 is still rewarded with 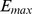 being the maximum energy bonus. If the restraint is satisfied, the full energy bonus is rewarded. Restraints that are only mildly violated (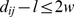 or 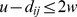) result in a decreased energy bonus. The contribution of the restraint falls back to zero in case of significant violation (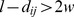 or 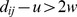). Note that the former case (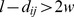) actually does not apply when modeling contact restraints with 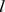 = 1.5 Å and 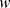 = 1.0. However, the potential is designed for general use and for other parameters (for example 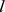 = 10 and 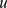 = 20) the 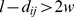 case is meaningful.

Source files that implement our scoring function are available on request.

## Results and Discussion

We evaluate the performance of EPC-map on the six test data sets described in Methods. We first evaluate our method on proteins of CASP10 and compare our results with the top methods from the CASP10 experiment. We then analyze the prediction performance of EPC-map on the remaining data sets. Then, we discuss how the performance of EPC-map varies with sequence alignment depth and protein chain length. Furthermore, we discuss the limitations of EPC-map and show that predicted contacts from EPC-map improve *ab initio* structure prediction.

We measure performance by evaluating the accuracy and coverage of the top scoring 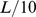, 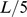 and 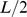 contacts from each prediction method, where 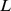 is the length of the protein. Because long-range contacts are of most value in structure modeling [45], our main discussion focuses on long-range contacts.

### Performance on test data sets

We first evaluate the contact prediction performance on proteins from the CASP10 experiment. This allows us to compare our approach with several methods that participated in CASP10. We downloaded the results of the CASP10 methods from the CASP10 website. EPC-map does not use any information from structural homologs. Therefore, it is appropriate to compare the performance of EPC-map with that of several other sequence-based methods.

We have selected the six methods that showed top performance in the CASP10 experiment [23], submitted predictions for all targets, and –to the best of our knowledge– did not use templates and/or server models for contact prediction.

We have chosen the following six methods: Group 305 (server name: IGB-Team, program name: CMAPpro) [21], Group 222 (server name: MULTICOM-construct, program name: DNCON) [7], Group 358 (server name RaptorX-Roll), Group 113 (server name: SAM-T08 Server, program name: SAM-T08) [47], Group 314 (server name: Proc
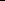
S4), Group 424 (MULTICOM-Novel, program name: NNcon) [16]. IGB-Team and MULTICOM-construct use deep networks to predict contacts. RaptorX-Roll uses a context-specific distance-based statistical potential [46], Proc_S4 uses random forests to predict contacts and is based on the original method that also participated in CASP9 [20]. SAM-T08 and MULTICOM-Novel are based on neural networks.

Additionally, we include PhyCMAP, a recent method based on random forests and physical constraints that has been shown to outperform current methods on the CASP10 dataset [44]. Furthermore, we include PSICOV [14] and GREMLIN [10] which predict contacts from evolutionary information. We run locally installed versions of PSICOV and GREMLIN; we use PhyCMAP by its web service. Finally, we evaluate contact prediction by occurrence frequencies on the decoys we generate, which we will refer to as Counting. This is similar to some of the most accurate contact predictors from the recent CASP experiments [22], [23]. The main difference is that decoy-based methods in the CASP setup use a consensus approach with decoys from several tertiary prediction servers that might use different energy functions, sampling methods and/or templates. The decoys from our Counting approach all stem from Rosetta *ab initio* generated decoys.

Unless stated otherwise in this section, we refer to the accuracy of the top scoring 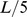 long-range contacts.

**Figure 3:**
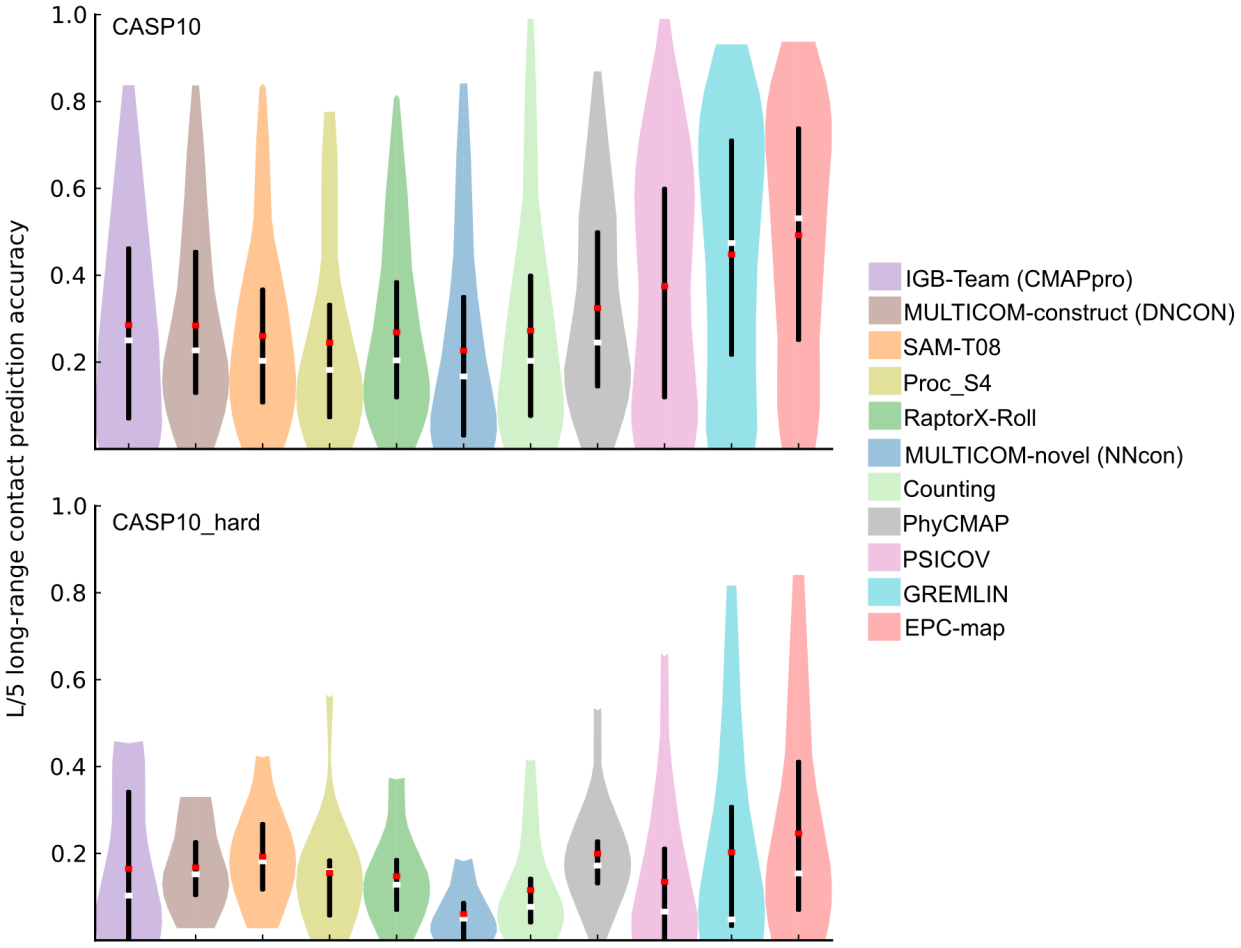
Prediction performance overview for the CASP10 and CASP10
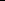
hard data sets. The figure shows the long-range contact prediction performance of the top scoring L/5 contacts. Different methods are shown as color coded violin plots. The lower and upper end of the black vertical bars in each violin denote the accuracy at the 25 and 75 percentile, respectively. White horizontal bars indicate the median, red horizontal bars the mean accuracy. The distribution of the prediction accuracies for individual proteins is indicated by the shape of the violin.

Figure 3 summarizes the long-range 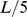 contact prediction performance on the CASP10 data set. Detailed information about the medium- and long-range performance on different 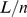 cutoffs is given in Tables S10 and S11 in File S1. EPC-map reaches a mean accuracy of 0.492, the second-best method (GREMLIN) reaches a mean accuracy of 0.448, followed by PhyCMAP with 0.325 mean accuracy. MULTICOM-construct(DNCON), the best performing method of the CASP10 experiment [23], has a mean accuracy of 0.285 on the CASP10 dataset. Thus, EPC-map is 4.4% more accurate than GREMLIN and 20.7% more accurate than MULTICOM-construct(DNCON) on the entire CASP10 dataset.

Of the 104 proteins in CASP10, 14 proteins contain domains that are classified by having free modeling or difficult free-modeling/template-based domains. For this kind of proteins, contact prediction is most useful because the structure cannot modeled by only using templates. For these difficult proteins (CASP10
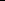
hard) the top performing methods of the CASP10 experiment predict contacts with 0.165–0.192 accuracy. GREMLIN and PhyCMAP are competitive on this dataset with 0.203 and 0.200 mean accuracy, respectively. EPC-map reaches 0.246 accuracy, improving on GREMLIN by 4.3% and being 5.4% more accurate than the best CASP10 method on this difficult data set.

Ideally, we would compare our method with the best methods of the CASP10 experiment on all test sets. Unfortunately, standalone versions of many of the best-performing CASP10 methods were not available to us at the time of this study and their server implementations are not designed for the evaluation of hundreds of proteins. Thus, for the remainder of this study, we only evaluate methods that are available as a standalone version or server that allows for high-throughput contact prediction. This includes NNcon, PhyCMAP, Counting, PSICOV and GREMLIN. PhyCMAP and GREMLIN perform on par or better than the top methods of the CASP10 experiment and can therefore be considered to be state of the art (see Figure 3). Therefore, comparing EPC-map with these methods provides a fair estimate of state-of-the-art performance. Note that for the remaining data sets, we use sequences and training proteins dated after CASP10 in EPC-map to evaluate the current capabilities of our method.

**Figure 4:**
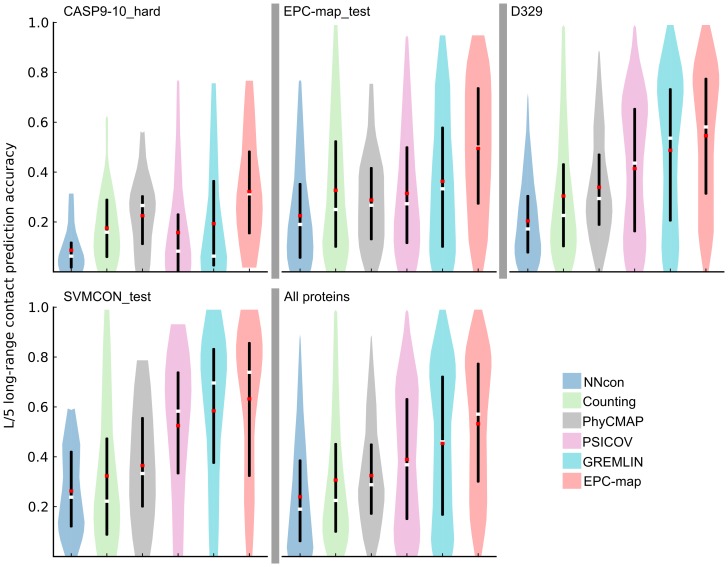
Prediction performance overview for the CASP9-10_hard, EPC-map_test, D329 and SVMCON_test data sets. The figure shows the long-range contact prediction performance of the top scoring L/5 contacts. Different methods are shown as color coded violin plots. The lower and upper end of the black vertical bars in each violin denote the accuracy at the 25 and 75 percentile, respectively. White horizontal bars indicate the median, red horizontal bars the mean accuracy. The distribution of the prediction accuracies for individual proteins is indicated by the shape of the violin. Data sets are sorted from difficult (CASP9-10_hard) to easy (SVMCON_test). The last panel shows the pooled results for all proteins from these data sets.

Figure 4 summarizes the long-range 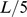 contact prediction accuracies, grouped by the remaining data sets (CASP9-10_hard, EPC-map_test, D329, SVMCON_test). For detailed analysis, refer to Tables S12–S15 in File S1.

**Figure 5:**
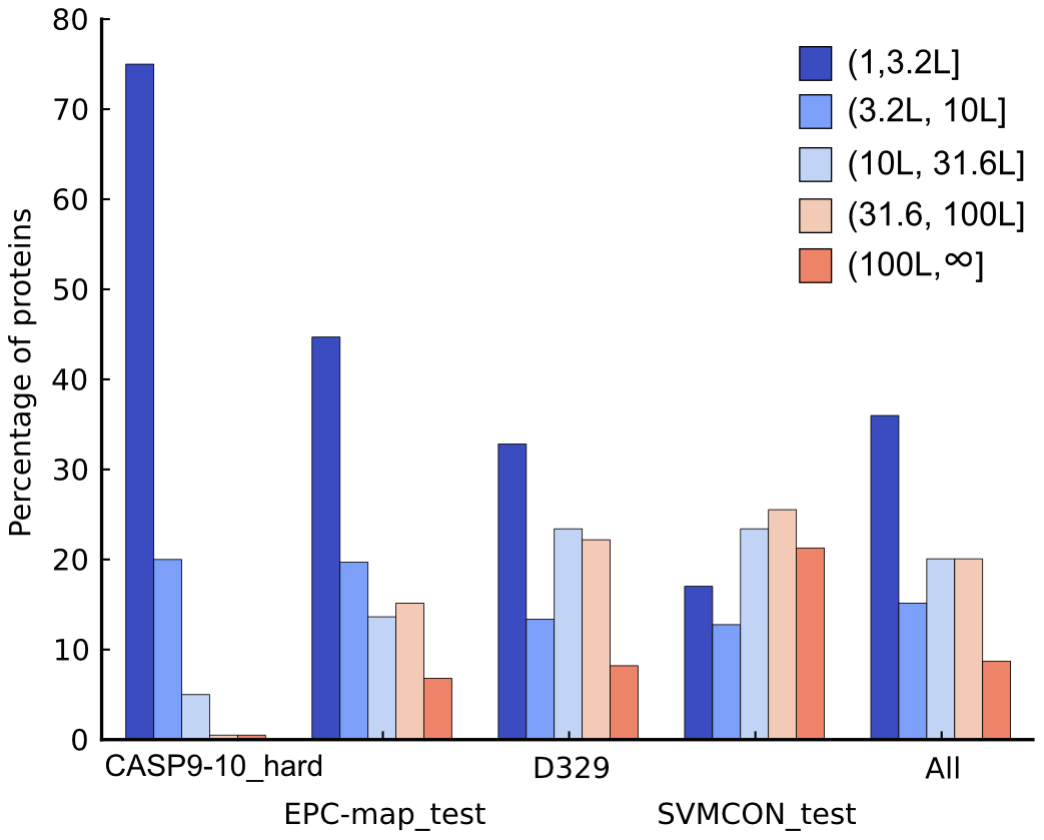
Alignment depth composition of the CASP9-10_hard, EPC-map_test, D329 and SVMCON_test data sets. Proteins are grouped into bins based on their number of sequences in the alignment. Colors correspond to a particular bin, from dark blue (few sequences) to red (many sequences). Data sets are sorted from difficult (CASP9-10_hard) to easy (SVMCON_test). The last panel shows the pooled results.

We structure our further discussion of prediction performance based on data set difficulty, as judged by the distribution of available sequences in the MSA, i.e. alignment depths (Figure 5). For the most difficult data set, CASP9-10_hard, EPC-map (mean accuracy 0.322) improves the mean prediction accuracy by 9.7% over the next best method (see Figure 4). Interestingly, neither the best structure-based method (Counting) nor the best method that uses evolutionary information (GREMLIN) delivers good results for this data set (mean accuracies of 0.173 and 0.193, respectively). However, the combination approach taken by EPC-map unlocks the potential of both, evolutionary and physicochemical information methods.

On the EPC-map_test data set, EPC-map (mean: 0.496, median: 0.5) performs on average 13.3% better than GREMLIN (mean: 0.363, median: 0.333), the second-best performing method. In this data set, 45% of the proteins have alignments with fewer than 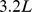 sequences. These proteins are difficult to predict with evolutionary methods [9], [10], showing that the approach taken by EPC-map is well suited for proteins with a low number of sequences.

For the easier data sets, D329 and SVMCON_test, the improvements are less pronounced but EPC-map still outperforms the second-best method by 5.9% and 4.8%, respectively. These two data sets contain many proteins with deep alignments, leading to the robust performance of methods relying on evolutionary sequence information. However, the additional physicochemical information leveraged by EPC-map leads to further performance improvements.

Averaged over 528 proteins from the CASP9-10_hard, EPC-map_test, D329 and SVMCON_test data sets, EPC-map reaches 53.2% mean accuracy and 57.1% accuracy at the median for top 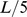 predicted long-range contacts. The second best is GREMLIN with 45.4% mean accuracy and 46% median accuracy. Thus, EPC-map improves the mean accuracy by 7.8% and the median accuracy by 11.1%. Additionally, predictions with 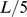 accuracy higher than 0.3 are more frequent for EPC-map (394 cases, 74%) then for GREMLIN (338 cases, 64%). We also find that EPC-map significantly improves the medium-range contact prediction accuracy (see Tables S10–S15 in File S1) in most cases.

EPC-map achieves superior performance by integrating the physicochemical information of the energy function of structure prediction with the evolutionary sequence information from multiple sequence alignments.

### Dependence of contact prediction accuracy on alignment depth and sequence length

In addition to the performance analysis on various data sets, we further analyzed the prediction performance as a function of other factors, such as alignment depth and sequence length. For this analysis, we used the proteins from the CASP9-10_hard, EPC-map_test, D329 and SVMCON_test data sets.

**Figure 6:**
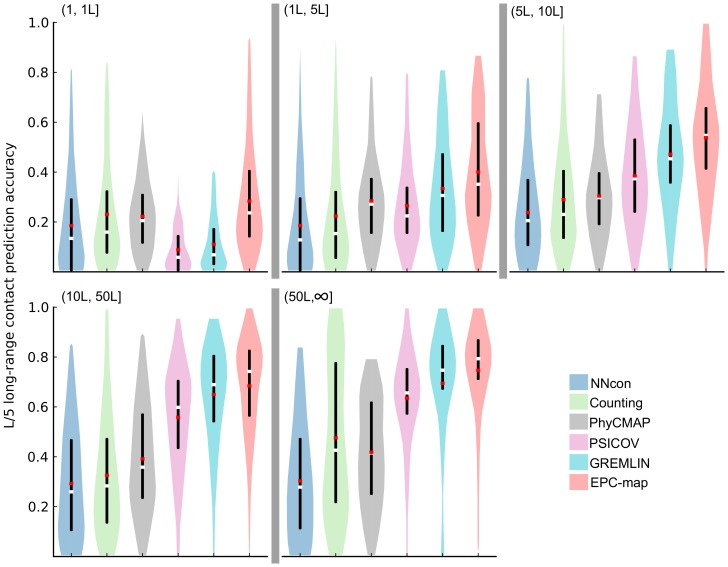
Prediction performance for proteins with increasing sequence alignment depth. Results are shown for all proteins pooled from the CASP9-10_hard, EPC-map_test, D329 and SVMCON_test data sets. Different methods are shown as color coded violin plots. The lower and upper end of the black vertical bars in each violin denote the accuracy at the 25 and 75 percentile, respectively. White horizontal bars indicate the median, red horizontal bars the mean accuracy. The distribution of the prediction accuracies for individual proteins is indicated by the shape of the violin. EPC-map is consistently more accurate than the other tested methods, regardless how many sequences are available.

Figure 6 shows the prediction performance with increasing alignment depth. The performance of all methods increases with the amount of available sequences. Evolutionary methods (PSICOV, GREMLIN), perform poorly in cases with less than 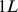 sequences, while being clearly superior to decoy-based (Counting) and machine-learning based methods (NNcon, PhyCMAP) in cases with more than 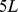 sequences. On the other hand, decoy-based and machine-learning based methods perform robustly in the (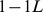] and (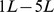] intervals, but do not benefit as much from more than 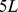 sequences as evolutionary methods. EPC-map improves prediction accuracy over the second best method, regardless how many sequences are available. This makes EPC-map a versatile approach to contact prediction that performs robustly for proteins with low and high numbers of homologous sequences.

Furthermore, we analyzed the performance of the SVM component and its contribution to the overall performance of the system. First, we analyze the performance of the single SVMs that are part of the SVM ensemble. The individual SVM classifiers reach accuracies between 0.267-0.287. Using an SVM ensemble improves the accuracy to 0.332 (see Table S16 in File S1). Thus the SVM ensemble improves prediction accuracy over the single SVMs. Furthermore, training five SVMs with small data subsamples facilitates faster training compared to a single SVM that uses all training data.

Second, we omit the SVM component and only combine Counting and GREMLIN scores. The 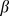 value is re-tuned in the same fashion as described in Methods. We find that the improvement by the SVM component is most pronounced when few sequences are available (see Table 2). In case of fewer than 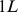 alignments, the SVM component improves the mean long-range 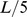 prediction accuracy by 3.4%. Our experiments show that physicochemical information is most helpful if insufficient sequences are available for sequence-based methods, effectively compensating their major shortcoming. We believe that combination of physicochemical and evolutionary information is an attractive route to advance the currently rapid evolving field of contact prediction.

**Table 2:** Contribution of the SVM component to contact prediction.

Method	Range	Acc(SE)/Cov[L/10]	Acc(SE)/Cov[L/5]	Acc(SE)/Cov[L/2]
120 proteins with (1, 1L] sequences				
with SVM	Long	0.335(0.023)/0.038	0.278(0.019)/0.062	0.205(0.014)/0.110
w/o SVM	Long	0.305(0.024)/0.035	0.244(0.019)/0.055	0.188(0.015)/0.102
102 proteins with (1L, 5L] sequences				
with SVM	Long	0.471(0.025)/0.045	0.395(0.022)/0.076	0.279(0.015)/0.134
w/o SVM	Long	0.475(0.026)/0.045	0.388(0.022)/0.073	0.280(0.016)/0.133
306 proteins with >5L sequences				
with SVM	Long	0.741(0.012)/0.071	0.678(0.012)/0.131	0.530(0.011)/0.253
w/o SVM	Long	0.739(0.012)/0.070	0.679(0.012)/0.131	0.528(0.011)/0.250

**Figure 7:**
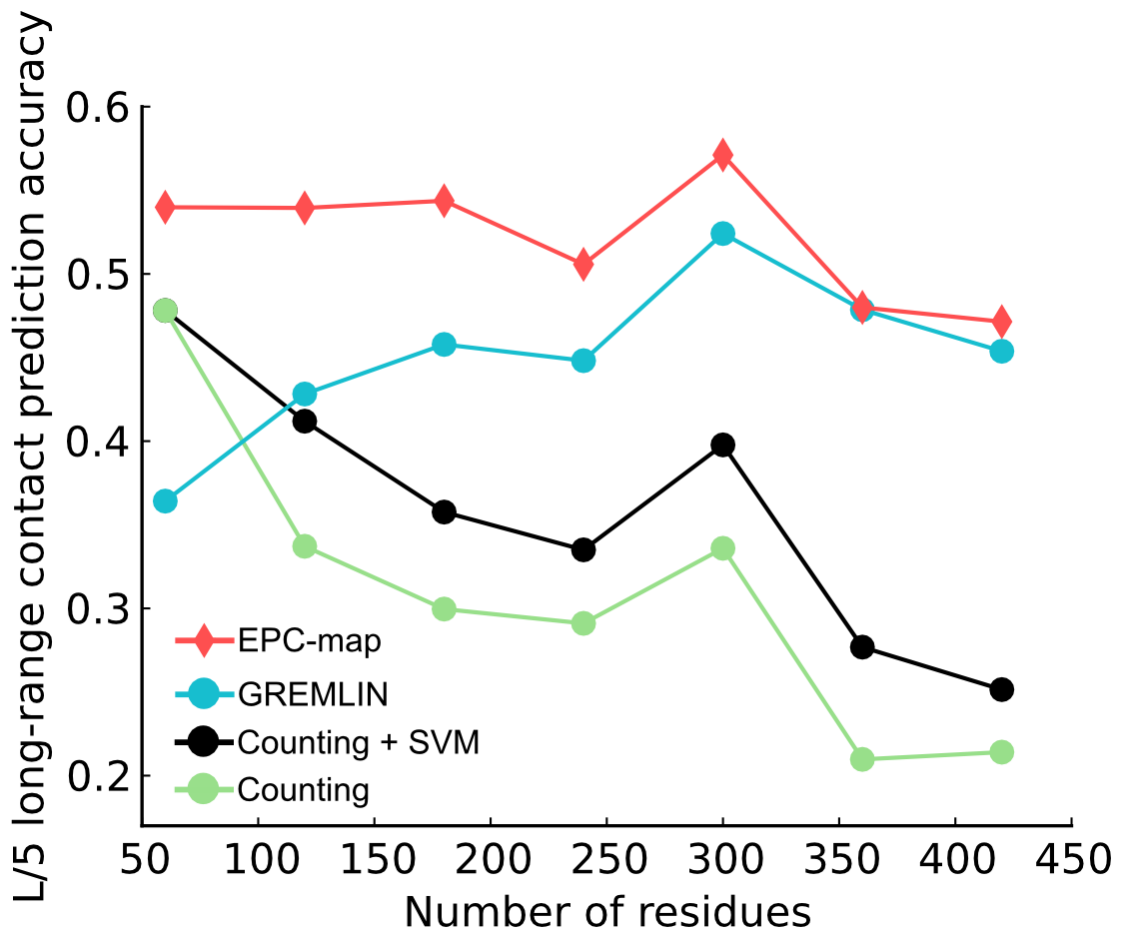
Dependence of prediction accuracy on sequence length. EPC-map is more accurate or on par with GREMLIN, irrespective of sequence length. The performance increase over GREMLIN is most pronounced for proteins smaller than 250 residues. Counting performs better on smaller proteins. The SVM component of EPC-map consistently improves the contact prediction from decoys over Counting by leveraging physicochemical information.

Naturally, any decoy based-method (such as EPC-map) depends on the quality of the generated decoys. Generating decoys for larger proteins is more difficult and they are likely poorer in quality. This might affect the prediction quality of decoy-dependent methods more than sequence-based methods. Figure 7 shows the prediction performance of EPC-map, GREMLIN, Counting and Counting +SVM (which is the SVM component from EPC-map) versus the sequence length of the proteins. The improvement in prediction accuracy by EPC-map is most pronounced for proteins smaller than 250 amino acids. For smaller proteins, Counting is performing better due to higher quality decoys. In part, this accounts for the good performance of EPC-map on shorter targets. However, the SVM component of EPC-map consistently improves pure decoy-based prediction over Counting by leveraging physicochemical information (see Figure 7). EPC-map is still more accurate than GREMLIN for longer proteins, but the performance improvement over GREMLIN is less pronounced, probably due to lower-quality decoys. Nevertheless, EPC-map is still ahead or on par with GREMLIN for larger proteins.

### Limitations of EPC-map

The computational most intense step of EPC-map is the generation of decoys for contact prediction. In the construction of our training and test sets, we limited the maximum length of the proteins to 150 amino acids to allow for faster training and testing. For proteins with 250 residues, Rosetta needs approximately ten minutes per decoy which results in 7 CPU days for 1000 decoys. We run Rosetta on a compute cluster with 100 nodes, thus we need about 100 minutes for decoy generation for a protein of this size. In contrast, feature generation and prediction with by the SVM ensemble is quite fast and takes only a couple of minutes on a single CPU. However, EPC-map is computationally much more intense than sequence-based methods.

On the one hand, this might render EPC-map unsuitable for some applications, such as proteome-wide analysis of protein contacts. On the other hand, decoy generation can be easily parallelized and run on low-cost commodity clusters with sufficient speed for many practical applications.

However, the main purpose of contact prediction is to aid *ab initio* tertiary structure prediction, which naturally requires substantial computational resources. Thus, the required computation power might already be available to many laboratories that work on *ab initio* structure prediction. For this application, EPC-map contacts might even save computational time needed in *ab initio* structure prediction by guiding conformational space search towards the native state. In any case, if the computational requirements exceed available resources, EPC-map predicted contacts can be obtained from our web service at http://compbio.robotics.tu-berlin.de/epc-map/.

### Improvement of *ab initio* structure prediction by using predicted contacts

The main purpose of contact prediction is to aid tertiary structure prediction. We tested the impact of including information from EPC-map predictions into *ab initio* Rosetta calculations for the 132 proteins from EPC-map
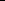
test. We model contacts as distance restraints using a bounded Lorentz function (see Methods for details). This function assigns an energy bonus to satisfied restraints. If a restraint is not satisfied, the energy bonus falls back to zero. This implies that restraints violated by a large margin are simply neglected, compensating the detrimental effect of false positive contact predictions. Example configuration files and commands of our contact-guided structure prediction setup are provided in the supporting information.

For each target, we generate 1000 decoys with contact restraints and 2000 without restraints. We use 2000 decoys in the second case to make a fair comparison in terms of sampling, since 1000 decoys were already used to predict contacts with EPC-map. We varied the number of predicted contacts to study the influence of the accuracy/coverage trade-off on structure prediction accuracy. We found that using 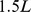 contacts gives the best performance.

**Figure 8:**
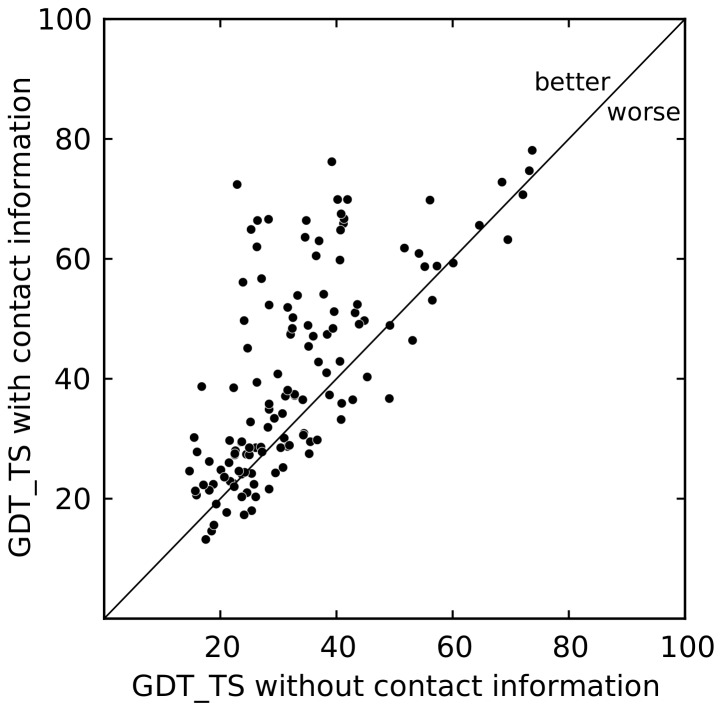
Comparison of *ab initio* structure prediction of 132 proteins from EPC-map_test with and without predicted contacts: each data point corresponds to the GDT_TS of the lowest-energy structure generated with and without the use of EPC-map predicted contacts. EPC-map increases the average prediction accuracy by 7.8% from 33.1 to 40.9 GDT_TS (paired Student's *t*-test *p*-value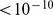).

The prediction improvements are depicted in Figure 8. We used the GDT
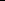
TS measure to quantify the quality of the predicted structures. The GDT
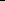
TS measure ranges from 0 if two structures are completely dissimilar to 100 for a perfect structural match. At a GDT_TS of 50 or more, a prediction is considered to capture the native topology. The average GDT_TS of contact-guided Rosetta increases to 40.9 compared to 33.1 using standard Rosetta (paired Student 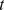-test 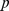-value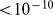), an absolute improvement of 7.8%. The GDT_TS increases by more than 10 for 41 of the 132 proteins. In 24 cases, the GDT_TS increase is higher than 20. In addition, for 21 proteins the GDT_TS transitions from well below 50 to 50 or higher. In these cases, the combination of EPC-map predicted contacts and Rosetta allows for the folding of proteins that could not be modeled with Rosetta alone. Thus, our results show that contact information from EPC-map readily enhances structure prediction performance.

We also notice that the prediction of one structure deteriorates by 10 GDT_TS when predicted contacts are used. In this case, most of the predicted contacts are wrong and mislead tertiary structure prediction.

**Figure 9:**
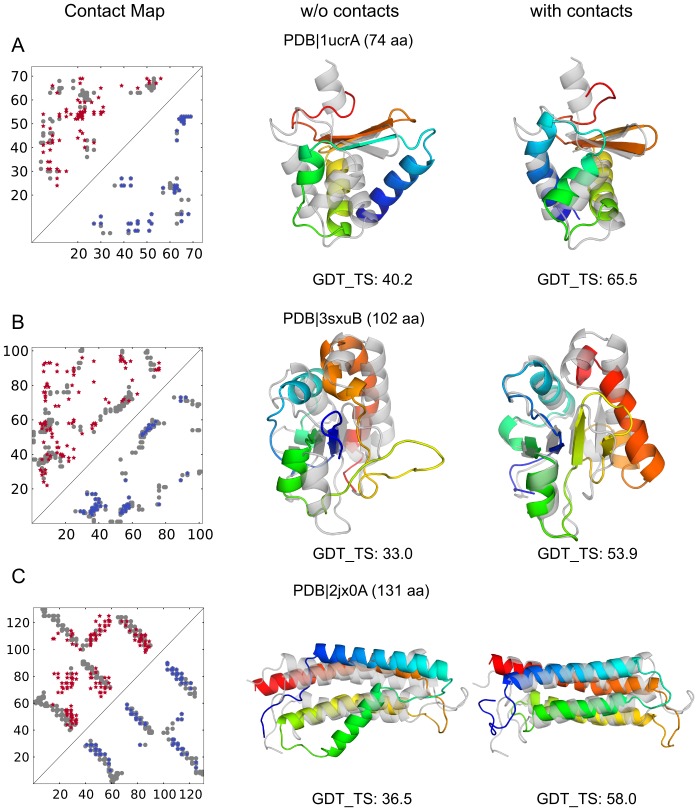
Tertiary structure prediction improvement of the dissimilatory sulfite reductase D (PDB
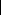
1ucrA), of the *E. coli* SSB-DNA polymerase III (PDB
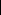
3sxuB) and of the GIT1 paxillin-binding domain (PDB
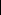
2jx0A). Contact maps show false positive predictions in the upper triangle (red), true positive predictions in the lower triangle (blue) and native contacts in grey. For the shown predictions, native structures are shown in grey and predicted structures are colored from N-terminus (blue) to C-terminus (red). The predictions correspond to the lowest-energy structure generated without use of contacts (middle column) and with EPC-map predicted contacts (right column).

Figure 9 shows three example proteins for which the combination of EPC-map and Rosetta yielded significant improvements in prediction accuracy. For these examples, only few homologous sequences are available (less then 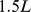). For each protein, we show the contact map obtained by EPC-map with true and false positives, the prediction of Rosetta without the inclusion of contact information, and the prediction based on contact information.

Without contacts, Rosetta fails to find the correct topology for the dissimilatory sulfite reductase D (PDB
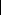
1ucrA, Figure 9A). The structure modeled with contacts from EPC-map resembles the native topology and has minor deviations in loop regions. However, the most C-terminal helix is still incorrectly modeled.

Rosetta predictions without contacts capture the general topology for the *E. coli* SSB-DNA polymerase III (PDB
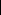
3sxuB, Figure 9B), but fail to arrange the 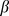-sheet topology. In contrast, with the help of the predicted contacts from EPC-map, more native-like *β*-sheet topologies can be sampled. The most C-terminal part of the structure is wrongly oriented by an incorrectly formed anti-parallel *β*-sheet. Nevertheless, the GDT_TS of the modeled structure increases from 33.0 to 53.9.

The most prominent feature of the GIT1 paxillin-binding domain(PDB
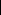
2jx0A, Figure 9C) is the four-helix bundle. In this case, Rosetta cannot model the topology correctly, especially of the second helix and fails to find the fine-tuned packing (GDT_TS 36.5). Predicted contacts from EPC-map guide Rosetta to the native topology (GDT_TS 58.0). However, the EPC-map guided prediction shows deviations from the native structure in the loop regions and N-terminus.

These experiments with contact-guided structure prediction demonstrate the potential of coupling EPC-map's contact prediction with structure prediction. The strategy of interleaving structure and contact prediction might be a promising future route to improve *ab initio* structure modeling.

## Conclusion

We presented EPC-map, a contact prediction method that achieves unprecedented prediction accuracy by combining evolutionary information from multiple-sequence alignments with physicochemical information from structure prediction methods. By combining two sources of information, our method improves prediction accuracy when compared to state-of-the-art algorithms. At the same time, we show that one source of information is able to compensate for the performance degradation induced by poor quality of the other source. This alleviates the main short-coming of popular evolution-based contact predictors, whose performance depends on the availability of many homologous sequences. We therefore believe that combining evolutionary and physicochemical information is an attractive route to improve contact prediction and reducing the need for deep alignments.

Key to the performance improvements achieved by our method is a graph-based representation of the characteristics of the local contact neighborhood to leverage physicochemical information. We use the graph-based representation to distill information about graph topology and label statistics into vector-based features. An SVM model is trained with these features to distinguish native from non-native contacts in *ab initio* decoys with unprecedented accuracy. We then combine this physicochemistry-based system with an evolutionary-based method to an approach that leads to substantial performance improvements over methods that only use a single source of information.

Using this strategy, EPC-map achieves 53.2% mean accuracy for top 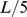 predicted long-range contacts over 528 proteins, 7.8% higher than the second-best method. Furthermore, EPC-map outperforms other top methods on proteins from CASP10. We showed that EPC-map displays improved performance regardless of the available alignment size, but is particulary effective if less than 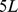 or even 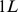 sequences are available. The predicted contacts improve *ab initio* structure prediction by guiding search in the conformational space towards the native state.

Our method is build to extract physicochemical contact information from structure decoys. One can expect that the quality of contact prediction increases as the quality of the generated decoys increases. Thus, we suggest that alternating between tertiary structure and contact prediction might be a promising route to incrementally increase the quality of contact information and of the resulting structural models.

## Supporting Information

File S1 **Text S1,** Graphs for modeling physicochemical context. **Text S2,** Features used and their generation. **Text S3,** Setup and example files for contact-guided Rosetta predictions. **Table S1,** Summary of node labels. **Table S2,** Summary of edge labels. **Table S3,** Pairwise features between contacting residues. **Table S4,** Graph topology features. **Table S5,** Graph spectrum features. **Table S6,** Single node features. **Table S7,** Node label statistics. **Table S8,** Edge label statistics. **Table S9,** Whole protein features. **Table S10,** Contact prediction performance of several methods on the CASP10 data set (104 proteins). **Table S11,** Contact prediction performance of several methods on the CASP10_hard data set (14 proteins). **Table S12,** Contact prediction performance of EPC-map, Counting, GREMLIN, PSICOV, PhyCMAP and NNcon on the CASP9-10_hard data set (20 proteins). **Table S13,** Contact prediction performance of EPC-map, Counting, GREMLIN, PSICOV, PhyCMAP and NNcon on the EPC-map_test data set (132 proteins). **Table S14,** Contact prediction performance of EPC-map, Counting, GREMLIN, PSICOV, PhyCMAP and NNcon on the D329 data set (329 proteins). **Table S15,** Contact prediction performance of EPC-map, Counting, GREMLIN, PSICOV, PhyCMAP and NNcon on the SVMCON_test data set (47 proteins). **Table S16,** Accuracies of the single SVM classifiers and the Ensemble SVM on 528 proteins from the CASP9-10_hard, EPC-map_test, D329 and SVMCON_test data sets.
(PDF)

File S2 **Dataset S1,** Proteins used for training of EPC-map. **Dataset S2,** Proteins used for validation of EPC-map. **Dataset S3,** Proteins from CASP10 used for validation of EPC-map. **Dataset S4,** Proteins from CASP10 containing at least one FM or FM/TBM domain. **Dataset S5,** Proteins from CASP9 and CASP10 containing only FM or FM/TBM domains. **Dataset S6,** The D329 data set contains proteins from literature that are used to access contact prediction performance. **Dataset S7,** The SVMCON_test data set contains proteins from literature that are used to access contact prediction performance.
(GZ) Click here for additional data file.
