# Supplementary material for: Combining Physicochemical and Evolutionary Information for Protein Contact Prediction
Source: PLoS One. 2014 Oct 22;9(10):e108438. doi: 10.1371/journal.pone.0108438 (PMC4206277; doi:10.1371/journal.pone.0108438)
Supplement: File S1 — Text S1, Graphs for modeling physicochemical context. Text S2, Features used and their generation. Text S3, Setup and example files for contact-guided Rosetta predictions. Table S1, Summary of node labels. Table S2, Summary of edge labels. Table S3, Pairwise features between contacting residues. Table S4, Graph topology features. Table S5, Graph spectrum features. Table S6, Single node features. Table S7, Node label statistics. Table S8, Edge label statistics. Table S9, Whole protein features. Table S10, Contact prediction performance of several methods on the CASP10 data set (104 proteins). Table S11, Contact prediction performance of several methods on the CASP10_hard data set (14 proteins). Table S12, Contact prediction performance of EPC-map, Counting, GREMLIN, PSICOV, PhyCMAP and NNcon on the CASP9-10_hard data set (20 proteins). Table S13, Contact prediction performance of EPC-map, Counting, GREMLIN, PSICOV, PhyCMAP and NNcon on the EPC-map_test data set (132 proteins). Table S14, Contact prediction performance of EPC-map, Counting, GREMLIN, PSICOV, PhyCMAP and NNcon on the D329 data set (329 proteins). Table S15, Contact prediction performance of EPC-map, Counting, GREMLIN, PSICOV, PhyCMAP and NNcon on the SVMCON_test data set (47 proteins). Table S16, Accuracies of the single SVM classifiers and the Ensemble SVM on 528 proteins from the CASP9-10_hard, EPC-map_test, D329 and SVMCON_test data sets. (PDF) [file pone.0108438.s001.pdf]

Combining Physicochemical and Evolutionary Information for Protein Contact Prediction
Supplementary Material

Text S1: Graphs for modeling physicochemical context

One of the main contributions of this work is the improvement of contact prediction with physicochemical information by incorporating the local contact context into prediction. This context is modeled as a graph, centered on the contact under consideration (see main text section 3.6 for graph definition). Nodes represent residues and edges represent contacts present in a decoy. The remainder of this section introduces the node and edge labels (summary in Tables S1 and S2), which are later used to derive features of contact graphs.

1.1. Node labels

Chemical type: Chemical properties of the residue are classified into four categories: non-polar, polar, acidic and basic.

Secondary structure: The secondary structure of the residue is classified into helix, sheet, turn or coil.

Table S1: Summary of node labels

Node label	Possible labels
Chemical type	Non-polar, polar, acidic, basic
Secondary structure	Helix, sheet, turn, coil
Solvent accessibility	Buried, exposed
Free solvation energy	Continuous value
Secondary structure length	Discrete value
Secondary structure 3D length	Continuous value
Secondary structure buried	Continuous value
Secondary structure exposed	Continuous value
Hydrogen bonding	Donor, acceptor, not involved
Distance to the centroid	Continuous value
Sequence conservation	Continuous value
Sequence neighborhood conservation	Continuous value

Solvent accessibility: Solvent accessibility of the residue is computed by POPS [1]. Residues with a relative solvent accessibility $> 25\%$ are labeled as exposed, residues with a relative solvent accessibility below or equal to this threshold are labeled as buried.

Free solvation energy: The free solvation energy of the residue, as computed by POPS.

Secondary structure length: This label represents the length of the secondary structure element, containing the residue of interest, in number of amino acids.

Secondary structure 3D length: The 3D length of the secondary structure element is measured by the distance (in Å) between the C_α atoms of the first residue and last residue belonging to the secondary structure element.

Secondary structure buried: The average number of buried residues in the residue's secondary structure element.

Secondary structure exposed: The average number of exposed residues in the residue's secondary structure element.

Hydrogen bonding: Donor, acceptor or not involved in hydrogen bonding.

Distance to the centroid: The distance of the residue to the centroid of the decoy in Å.

Sequence conservation: The conservation of the sequence at the residue position in the multiple-sequence alignment. The conservation has been computed as in [2, 3].

Sequence neighborhood conservation: The conservation of the sequence neighborhood was characterized for sequence positions $i-3$, $i-2$ $i-1$, $i+3$, $i+2$ $i+1$ as in [2, 3].

1.2. Edge labels

Table S2: Summary of edge labels

Edge label	Possible labels
Contact potential	Continuous value
3D distance	Continuous value
Sequence separation	Discrete value
Mutual information	Continuous value

Contact potential: The contact potential, introduced by Li et al. [4] describes the likelihood of observing a contact between particular types of amino acids (for example, ASP-GLU) in a high-resolution set of crystal structures.

3D distance: Distance between C_β atoms of the contacting residues (C_α for glycine) in the decoy.

Sequence separation: The sequence separation between contacting residues in number of amino acids.

Mutual information: The mutual information in the multiple-sequence alignment between positions i and j.

Text S2: Features used and their generation

In order to learn to distinguish native from non-native contacts, we designed a number of features to capture different physicochemical context properties of contacting residues. Each feature maps its corresponding property to a number of binary and/or continuously valued inputs for an SVM. The resulting input vector is the concatenation of the inputs generated by the individual features.

We broadly classify our features into eight groups: Pairwise, graph topology, graph spectrum, single node, node label statistics, edge label statistics and whole protein features. An overview of all feature classes can be found in the main document (Table 1). In this section, we give a detailed description of each feature and how it is calculated.

Distinct types of graph features are discussed in each of the following tables. Additionally, we provide a detailed description for any feature that is not self-explanatory.

2.1. Pairwise residue features

Pairwise features are used to capture any physicochemical or structural properties of the contacting residue pairs. Categorical features of the contacting residues i and j are encoded by a series of binary inputs. For instance, a property could be described by two states s_1 and s_2. Then, the bit vector would be $[1, 0, 0]^T$, if both residues are in s_1, $[0, 1, 0]^T$ if one residue is in s_1 and the other in s_2 and $[0, 0, 1]^T$ if both residues are in state s_2. Unless otherwise stated, we apply this encoding to all features with discrete states. An overview over the pairwise features is given in Table S3.

Chemical type: Chemical properties of the residues are classified into four categories: non-polar, polar, acidic and basic. Thus, 10 combinations of residue types are possible

Table S3: Pairwise features between contacting residues i and j

Feature	Description	Number of inputs
Chemical type	Chemical type of the contacting amino acids: non-polar, polar, acidic, basic	10[a]
Secondary structure	Secondary structure of the contacting amino acids: helix, sheet, turn, coil	10[a]
Solvent accessibility	Solvent accessibility of the contacting amino acids: exposed, buried	3[a]
Hydrogen bonding	Hydrogen bonding state of the contacting amino acids: donor, acceptor	2[a]
Sequence separation	Sequence separation encoded in 17 bins	17[a]
Sequence separation from N/C-terminus	Distance in amino acids between i and N-terminus; j and C-terminus	2
Contact potential	Contact potential from Li et al. [4]	1
Distance	3D distance between i and j	1
Mutual information	Sequence mutual information	1
Ensemble distance	Mean distance and standard deviation of i and j in ensemble if $d_{ij} \leq$12 Å	2
Total inputs		49

[a]Binary inputs

for a contact pair [5] (10 inputs).

Secondary structure: The secondary structure of the residues is classified into helix, sheet, turn or coil. The secondary structure state in the decoys is determined with STRIDE [6] (10 inputs).

Solvent accessibility: The solvent accessibility of residues is classified into solvent exposed or buried (see section 1.1) (3 inputs).

Hydrogen bonding: This feature checks whether the contacting residues are involved in hydrogen bonding and whether they act as a donor or acceptor (2 inputs).

Sequence separation: The sequence separation between the contacting residues is encoded by 17 binary inputs (12-13, 14-15, 16-17, 18-19, 20-21, 22-23, 24-28, 29-32, 33-36, 37-40, 41-44, 45-48, 49-52, 53-57, 58-62, 63-67, $<$68) (17 inputs).

Sequence separation from N/C-terminus: The sequence separation from the N-terminus to the more N-terminal residue and the sequence separation from the C-terminus to the more C-terminal residue, respectively (2 inputs).

Mutual information: The mutual information in the multiple-sequence alignment between positions i and j (1 input).

Ensemble distance: For this feature, we compute the average and the standard deviation of the distance between the contacting residues across all decoy structures in the ensemble, where the distance is $\leq$12 Å (2 inputs).

2.2. Graph features

We hypothesize that native and non-native contacts differ in their neighborhood to some degree. Thus, measuring the similarity between graphs that model the neighborhood of contacting residues should help us to differentiate native and non-native contacts. We accomplish this by using graph features to describe the characteristics of a graph.

Graph features are topological features and node/edge label statistics extracted from the graph. This approach was introduced in [7] and has been shown to be competitive with other state-of-the-art graph kernel approaches in graph classification tasks. In addition to some of the described features in [7], we design a number of domain-specific features for contact prediction. The features encode graph properties into vectors which can be compared by the standard kernel functions such as the radial basis function. This simple representation of the graph by a feature vector allows for easy integration with existing

software packages. Many of the graph-based calculations in this work are carried out with the Python library NetworkX [8].

Graph features are separately extracted for the shared and immediate neighborhood graphs. Thus, each graph feature is present two times in the final input vector.

2.2.1. Graph topology

Graph topology features describe different topological properties of the underlying contact graph (Table S4).

Table S4: Graph topology features

Feature	Description	Number of inputs
Number of nodes	Number of nodes in the graph	1
Number of edges	Number of edges in the graph	1
Average degree centrality	See text	1
Average closeness centrality	See text	1
Average betweenness centrality	See text	1
Average eccentricity	See text	1
Graph radius	See text	1
Graph diameter	See text	1
Number of end points	See text	1
Average clustering coefficient	See text	1
Total inputs		10

Average degree centrality: The degree centrality for a node is the fraction of nodes in the graph connected to the node. We take the average over all nodes to measure the average degree centrality of the graph. This metric can be viewed as a measure of packing density in the graph under consideration, with tightly packed regions having large

average degree centrality values (1 input).

Average closeness centrality: The average closeness centrality for one node is the reciprocal average path length from the node to all other nodes. The average over the closeness centralities of all nodes is then used as a feature for the graph. We interpret this as another measure for packing, in particular as a measure for the packing density of the residues in the contact graph (1 input).

Average betweenness centrality: The betweenness centrality of a node is the number of shortest paths from the set of all-pair shortest paths of the graph passing through the node. The average betweenness centrality of the graph is the average over all nodes. This measures the number of short-cuts present in the contact graph without the need for walking over the entire protein chain. In our view, this is related to the loss in conformational entropy caused by the formation of the contact network (1 input).

Average eccentricity: The eccentricity is the length of the longest of all all-pair shortest paths that pass through a node. Average eccentricity is the average over all nodes (1 input).

Graph radius: The graph radius is defined as the smallest eccentricity value of all nodes in the graph (1 input).

Graph diameter: The graph diameter is defined as the largest eccentricity value of all nodes in the graph (1 input).

Number of end points: Number of nodes with degree one (1 input).

Average clustering coefficient: The clustering coefficient of a node is the ratio of actual edges between neighbors of a node to the number of possible edges between them. The average clustering coefficient is the average over all nodes (1 input).

2.2.2. Graph spectrum

Table S5: Graph spectrum features

Feature	Description	Number of inputs
Largest eigenvalue	Largest eigenvalue	1
Second largest eigenvalue	Second largest eigenvalue	1
Number of different eigenvalues	Number of different eigenvalues	1
Sum of eigenvalues	Trace of the adjacency matrix	1
Energy	Sum of squared eigenvalues	1
Total inputs		5

Graph spectrum features are extracted from the adjacency matrix of the graph, which reflects the connectivity of the graph. Spectrum features are based on the eigenvalues of the adjacency matrix (Table S5).

Number of different eigenvalues: The number of eigenvalues with different values in the adjacency matrix of the graph (1 input).

Sum of eigenvalues: Sum of all eigenvalues of the adjacency matrix. This is equivalent to the trace of the adjacency matrix (1 input).

Energy: The energy of the graph is the sum of all squared eigenvalues of the adjacency matrix (1 input).

2.2.3. Single node features

Single node features describe some topological properties of the residues i and j due to their embedding in the contact network. The features are calculated for residues i and j separately. A summary of single node features is given in Table S6.

Table S6: Single node features

Feature	Description	Number of inputs
Degree	Node degree in the graph	1
Closeness centrality	Reciprocal average path length from the node to all other nodes	1
Betweenness centrality	Average number of shortest paths that pass through the node	1
Sequence conservation	Conservation of residue position in multiple sequence alignment	1
Sequence neighborhood conservation	Conservation of neighboring residues in multiple-sequence alignment	1
Total inputs		5

2.2.4. Node label statistics

Node label statistics are used to describe the distribution of node labels in the graph (Table S7). If the node label has a discrete value (such as chemical type), the distribution is simply the counts over the distinct node labels in the graph. Continuous values, such as the solvation energy of a residue, are discretized into multiple bins. The distribution is then the number of counts of the continuous labels that fall into each bin.

The node label statistics are separately calculated for each type of label. Examples of label types are the secondary structure and the solvent accessibility of the nodes.

Chemical type: Number of nodes with polar, non-polar, basic or acidic labels in the graph. This feature measures the distribution of the chemical types of amino acids in the graph (4 inputs).

Secondary structure: Number of nodes with helix, sheet, turn and coil labels (4 inputs).

Secondary structure length: The average length (in amino acids) of the secondary structure elements in the graph. This feature, as well as any other secondary structure

Table S7: Node label statistics

Feature	Description	Number of inputs
Chemical type	Number of polar, non-polar, acidic, basic labels	4
Secondary structure	Number of nodes with helix, sheet, turn, coil labels	4
Secondary structure length	Average length of secondary structure element in amino acids	4
Secondary structure 3D length	Average 3D length of secondary structure element	4
Secondary structure buried	Average number of buried residues in secondary structure element	4
Secondary structure exposed	Average number of exposed residues in secondary structure element	4
Solvent accessibility	Number of exposed/buried nodes	2
Hydrogen bonding	Number of nodes that act as donor, acceptor or do not form hydrogen bonds	3
Average solvation energy	Average free solvation energy	1
Solvation energy distribution	4-bin distribution of free solvation energy	4
Label entropy	Entropy of the labels	3
Neighborhood impurity degree	Average number of neighbors with different labels	3
Distance to centroid	Average distance of nodes to the centroid	1
Sequence conservation	Average sequence conservation of nodes	1
Sequence neighborhood conservation	Average sequence neighborhood conservation of nodes	1
Total inputs		43

description feature, is calculated separately for each secondary structure type (4 inputs).

Secondary structure 3D length: The average length in 3D of the secondary structure elements in the graph (4 inputs).

Secondary structure buried: Average number of buried residues in the secondary structure elements of a specific type (4 inputs).

Secondary structure exposed: Average number of exposed residues in the secondary structure elements of a specific type (4 inputs).

Solvent accessibility: Number of exposed and buried nodes in the graph (2 inputs).

Hydrogen bonding: Number of nodes involved in hydrogen bonding as a donor, acceptor or do not form any hydrogen bonds (3 inputs).

Average solvation energy: The average of all free solvation energies in the graph (1 input).

Solvation energy distribution: The 4-bin distribution of all free solvation energies in the graph (4 inputs).

Label entropy: Calculates the entropy of one class of labels. The label entropy is calculated separately for chemical type, secondary structure and solvent accessibility (3 inputs).

Neighborhood impurity degree: Calculates the number of neighbors of a node that have a different label than the node. The feature value for the graph is the averaged impurity degree of all nodes. This feature is also separately calculated for chemical type, secondary structure and solvent accessibility (3 inputs).

2.2.5. Edge label statistics

Edge label statistics describe the distribution of edge labels in the graph in the same fashion as node labels (Table S8).

Table S8: Edge label statistics

Feature	Description	Number of inputs
Link impurity	Number of edges connecting two nodes with different labels	3
Mutual information distribution	5-bin distribution of mutual information	5
Cumulative mutual information	Cumulative mutual information over all edges	1
Contact potential	3-bin distribution of contact potential	3
Total inputs		12

Link impurity: Calculates the fraction of edges, connecting two nodes with different node labels. This feature is separately calculated for chemical type, secondary structure and solvent accessibility (3 inputs).

Mutual information distribution: To calculate the mutual information distribution of the graph, we calculate the number of edges that are formed by nodes with different ranges of sequence separation (adjacent, 2-6, 7-11, 12-23, $>$24). This 5-bin distribution describes the mutual information distribution of the graph (5 inputs).

Contact potential: The potential of Li et al. [4] is binned into edges with low (smaller than 0.1), medium (between 0.1 and 0.3) or high (larger than 0.3) contact potential. The distribution of contact potential is given by the number of edges with contact potential that fall into each bin (3 inputs).

2.3. Whole protein features

Whole protein features capture global properties of the protein (Table S9).

Table S9: Whole protein features

Feature	Description	Number of inputs
Amino acid composition	Occurrence of each amino acid in the protein	20
Secondary structure composition	Occurrence of secondary structure in decoy	4
Length class	Binned length of the protein	5[a]
Total inputs		29

[a]Binary inputs

Amino acid composition: The composition of amino acids in the protein (20 inputs).

Secondary structure composition: The composition of secondary structures (helix, sheet, turn, coil) in the decoy (4 inputs).

Length class: The length of the protein in amino acids is binned into 5 categorical inputs ($<$60, 60-89, 90-119, 120-149, $>$150) (5 inputs).

In total, this results in an input vector of length 228. Note that all features defined on graphs are evaluated for the shared and the immediate neighborhood graph separately and therefore are present two times in the final input vector.

Text S3: Setup and example files for contact-guided Rosetta predictions

The following section supplies flag file and constraint file examples that we used to perform contact-guided predictions with Rosetta.

Example flag file:

```
-abinitio::fastrelax
-in::file::fasta 2kjpA.fasta
-in::file::frag3 2kjpA.200.3mers
-in::file::frag9 2kjpA.200.9mers
-constraints::cst_file 2kjpA_contact_constraints.txt
-in::path::database <path_to_rosetta_database>
-out::path .
-out::nstruct 1000
-mute core.chemical core protocols core.util.prof
```

Example restraints:

```
AtomPair CB 26 CB 54 LORENTZ 1.5 8.0 1.5
AtomPair CB 22 CB 57 LORENTZ 1.5 8.0 1.5
AtomPair CB 52 CB 65 LORENTZ 1.5 8.0 1.5
```

Rosetta is then executed by using the command: `AbinitioRelax.linuxgccrelease @flags`

Text S4: Supplementary tables

Table S10: Contact prediction performance of several methods on the CASP10 data set (104 proteins)

Method	Range	Acc^a(SE^b)/Cov^c[L/10]	Acc^a(SE^b)/Cov^c[L/5]	Acc^a(SE^b)/Cov^c[L/2]
EPC-map	Long	0.561(0.030)/0.064	0.492(0.028)/0.105	0.378(0.024)/0.188
IGB-Team (CMAPpro)	Long	0.338(0.027)/0.033	0.285(0.023)/0.057	0.208(0.017)/0.100
MULTICOM-construct (DNcon)	Long	0.327(0.025)/0.030	0.285(0.021)/0.053	0.215(0.015)/0.101
SAM-T08	Long	0.288(0.024)/0.028	0.260(0.020)/0.050	0.202(0.016)/0.093
ProC_S4	Long	0.285(0.026)/0.030	0.245(0.022)/0.048	0.183(0.015)/0.091
RaptorX-Roll	Long	0.308(0.024)/0.032	0.269(0.020)/0.053	0.211(0.016)/0.097
MULTICOM-novel (NNcon)	Long	0.212(0.021)/0.020	0.167(0.017)/0.031	0.120(0.011)/0.055
Counting	Long	0.338(0.030)/0.039	0.272(0.025)/0.059	0.184(0.016)/0.096
GREMLIN	Long	0.498(0.031)/0.047	0.448(0.029)/0.082	0.341(0.025)/0.153
PSICOV	Long	0.447(0.031)/0.036	0.375(0.028)/0.061	0.284(0.023)/0.117
PhyCMAP	Long	0.365(0.026)/0.031	0.325(0.022)/0.059	0.246(0.016)/0.106
EPC-map	Medium	0.648(0.026)/0.159	0.537(0.025)/0.258	0.362(0.020)/0.406
IGB-Team (CMAPpro)	Medium	0.429(0.026)/0.105	0.361(0.022)/0.173	0.263(0.015)/0.301
MULTICOM-construct (DNcon)	Medium	0.454(0.026)/0.106	0.377(0.021)/0.177	0.276(0.016)/0.313
SAM-T08	Medium	0.381(0.021)/0.090	0.322(0.017)/0.153	0.237(0.013)/0.270
ProC_S4	Medium	0.447(0.024)/0.106	0.370(0.020)/0.176	0.272(0.014)/0.316
RaptorX-Roll	Medium	0.464(0.022)/0.109	0.393(0.019)/0.181	0.301(0.015)/0.300
MULTICOM-novel (NNcon)	Medium	0.400(0.027)/0.093	0.329(0.022)/0.148	0.248(0.016)/0.257
Counting	Medium	0.543(0.031)/0.121	0.453(0.027)/0.201	0.308(0.019)/0.326
GREMLIN	Medium	0.473(0.029)/0.110	0.380(0.026)/0.171	0.242(0.019)/0.256
PSICOV	Medium	0.429(0.030)/0.088	0.345(0.026)/0.137	0.229(0.019)/0.215
PhyCMAP	Medium	0.474(0.023)/0.103	0.418(0.021)/0.179	0.309(0.016)/0.321

^{a}Accuracy
^{b}Standard error
^{c}Coverage

Table S11: Contact prediction performance of several methods on the CASP10_hard data set (14 proteins)

Method	Range	Acc[a](SE[b])/Cov[c][L/10]	Acc[a](SE[b])/Cov[c][L/5]	Acc[a](SE[b])/Cov[c][L/2]
EPC-map	Long	0.280(0.081)/0.026	0.246(0.068)/0.046	0.169(0.045)/0.076
IGB-Team (CMAPpro)	Long	0.201(0.062)/0.015	0.165(0.047)/0.025	0.126(0.032)/0.049
MULTICOM-construct (DNcon)	Long	0.192(0.036)/0.015	0.168(0.027)/0.028	0.130(0.018)/0.054
SAM-T08	Long	0.222(0.041)/0.018	0.192(0.032)/0.033	0.147(0.024)/0.062
ProC_S4	Long	0.170(0.050)/0.013	0.155(0.037)/0.025	0.115(0.022)/0.047
RaptorX-Roll	Long	0.130(0.033)/0.011	0.147(0.029)/0.027	0.130(0.025)/0.060
MULTICOM-novel (NNcon)	Long	0.102(0.029)/0.008	0.074(0.018)/0.012	0.051(0.011)/0.020
Counting	Long	0.148(0.041)/0.013	0.116(0.033)/0.022	0.084(0.019)/0.039
GREMLIN	Long	0.257(0.082)/0.022	0.203(0.068)/0.034	0.155(0.050)/0.064
PSICOV	Long	0.191(0.071)/0.016	0.135(0.052)/0.022	0.104(0.036)/0.042
PhyCMAP	Long	0.240(0.049)/0.020	0.200(0.034)/0.033	0.154(0.026)/0.064
EPC-map	Medium	0.438(0.089)/0.110	0.344(0.074)/0.171	0.238(0.055)/0.263
IGB-Team (CMAPpro)	Medium	0.317(0.079)/0.085	0.258(0.060)/0.136	0.189(0.038)/0.229
MULTICOM-construct (DNcon)	Medium	0.348(0.068)/0.079	0.279(0.053)/0.121	0.216(0.041)/0.237
SAM-T08	Medium	0.300(0.061)/0.074	0.286(0.048)/0.147	0.203(0.039)/0.239
ProC_S4	Medium	0.286(0.054)/0.070	0.242(0.042)/0.117	0.185(0.030)/0.220
RaptorX-Roll	Medium	0.342(0.075)/0.082	0.283(0.057)/0.135	0.222(0.040)/0.233
MULTICOM-novel (NNcon)	Medium	0.278(0.070)/0.067	0.232(0.051)/0.108	0.176(0.034)/0.192
Counting	Medium	0.360(0.083)/0.088	0.311(0.064)/0.151	0.211(0.041)/0.239
GREMLIN	Medium	0.270(0.084)/0.060	0.213(0.064)/0.090	0.156(0.056)/0.137
PSICOV	Medium	0.277(0.091)/0.061	0.216(0.078)/0.085	0.156(0.062)/0.136
PhyCMAP	Medium	0.346(0.077)/0.077	0.303(0.062)/0.136	0.232(0.041)/0.249

[a]Accuracy
[b]Standard error
[c]Coverage

Table S12: Contact prediction performance of EPC-map, Counting, GREMLIN, PSICOV, PhyCMAP and NNcon on the CASP9-10_hard data set (20 proteins)

Method	Range	Acc[a](SE[b])/Cov[c][L/10]	Acc[a](SE[b])/Cov[c][L/5]	Acc[a](SE[b])/Cov[c][L/2]
EPC-map	Long	0.414(0.064)/0.046	0.322(0.049)/0.072	0.222(0.031)/0.122
Counting	Long	0.246(0.055)/0.028	0.176(0.034)/0.043	0.120(0.017)/0.072
GREMLIN	Long	0.230(0.062)/0.022	0.193(0.053)/0.038	0.134(0.038)/0.063
PSICOV	Long	0.192(0.054)/0.018	0.157(0.048)/0.030	0.111(0.034)/0.052
PhyCMAP	Long	0.277(0.044)/0.029	0.225(0.034)/0.046	0.169(0.023)/0.088
NNcon	Long	0.097(0.031)/0.008	0.089(0.021)/0.016	0.080(0.021)/0.041
EPC-map	Medium	0.445(0.076)/0.146	0.343(0.053)/0.223	0.216(0.033)/0.332
Counting	Medium	0.407(0.075)/0.138	0.312(0.059)/0.200	0.196(0.036)/0.301
GREMLIN	Medium	0.166(0.038)/0.055	0.120(0.027)/0.081	0.080(0.015)/0.140
PSICOV	Medium	0.150(0.040)/0.043	0.114(0.033)/0.063	0.086(0.027)/0.105
PhyCMAP	Medium	0.311(0.055)/0.087	0.273(0.047)/0.153	0.186(0.027)/0.269
NNcon	Medium	0.158(0.042)/0.049	0.141(0.034)/0.085	0.122(0.026)/0.173

[a]Accuracy
[b]Standard error
[c]Coverage

Table S13: Contact prediction performance of EPC-map, Counting, GREMLIN, PSICOV, PhyCMAP and NNcon on the EPC-map_test data set (132 proteins)

Method	Range	Acc[a](SE[b])/Cov[c][L/10]	Acc[a](SE[b])/Cov[c][L/5]	Acc[a](SE[b])/Cov[c][L/2]
EPC-map	Long	0.553(0.026)/0.059	0.496(0.023)/0.109	0.376(0.019)/0.205
Counting	Long	0.378(0.028)/0.042	0.327(0.024)/0.076	0.263(0.018)/0.150
GREMLIN	Long	0.426(0.027)/0.044	0.363(0.024)/0.077	0.268(0.019)/0.139
PSICOV	Long	0.383(0.026)/0.040	0.315(0.021)/0.066	0.218(0.016)/0.114
PhyCMAP	Long	0.320(0.020)/0.033	0.288(0.016)/0.061	0.228(0.012)/0.123
NNcon	Long	0.251(0.022)/0.024	0.225(0.017)/0.045	0.183(0.012)/0.095
EPC-map	Medium	0.632(0.023)/0.159	0.523(0.021)/0.264	0.358(0.016)/0.437
Counting	Medium	0.577(0.026)/0.147	0.475(0.023)/0.245	0.322(0.016)/0.400
GREMLIN	Medium	0.408(0.025)/0.100	0.313(0.021)/0.152	0.197(0.013)/0.234
PSICOV	Medium	0.339(0.024)/0.082	0.226(0.018)/0.128	0.173(0.012)/0.203
PhyCMAP	Medium	0.440(0.023)/0.106	0.363(0.018)/0.178	0.273(0.013)/0.329
NNcon	Medium	0.335(0.024)/0.079	0.296(0.020)/0.138	0.209(0.013)/0.246

[a]Accuracy
[b]Standard error
[c]Coverage

Table S14: Contact prediction performance of EPC-map, Counting, GREMLIN, PSICOV, PhyCMAP and NNcon on the D329 data set (329 proteins)

Method	Range	Acc[a](SE[b])/Cov[c][L/10]	Acc[a](SE[b])/Cov[c][L/5]	Acc[a](SE[b])/Cov[c][L/2]
EPC-map	Long	0.613(0.016)/0.057	0.546(0.015)/0.102	0.421(0.013)/0.195
Counting	Long	0.363(0.017)/0.036	0.304(0.014)/0.061	0.219(0.010)/0.107
GREMLIN	Long	0.545(0.017)/0.047	0.487(0.016)/0.086	0.368(0.013)/0.162
PSICOV	Long	0.485(0.017)/0.041	0.414(0.015)/0.072	0.293(0.011)/0.128
PhyCMAP	Long	0.393(0.014)/0.033	0.339(0.011)/0.059	0.256(0.008)/0.110
NNcon	Long	0.236(0.011)/0.020	0.204(0.009)/0.035	0.156(0.006)/0.067
DNCON[d]	Long	-	0.329(0.037)/0.066	-
EPC-map	Medium	0.663(0.014)/0.173	0.563(0.013)/0.287	0.380(0.011)/0.464
Counting	Medium	0.578(0.016)/0.148	0.476(0.014)/0.241	0.323(0.010)/0.395
GREMLIN	Medium	0.468(0.017)/0.115	0.369(0.014)/0.179	0.230(0.009)/0.274
PSICOV	Medium	0.411(0.016)/0.100	0.320(0.013)/0.152	0.202(0.008)/0.237
PhyCMAP	Medium	0.471(0.013)/0.113	0.406(0.011)/0.196	0.295(0.008)/0.356
NNcon	Medium	0.380(0.015)/0.087	0.324(0.012)/0.149	0.231(0.008)/0.266
DNCON[d]	Long	-	0.427(0.036)/0.192	-

[a]Accuracy
[b]Standard error
[c]Coverage
[d]Values reported in the original paper of DNCON [9]

Table S15: Contact prediction performance of EPC-map, Counting, GREMLIN, PSICOV, PhyCMAP and NNcon on the SVMCON_test data set (47 proteins)

Method	Range	Acc[a](SE[b])/Cov[c][L/10]	Acc[a](SE[b])/Cov[c][L/5]	Acc[a](SE[b])/Cov[c][L/2]
EPC-map	Long	0.679(0.044)/0.067	0.632(0.044)/0.127	0.482(0.036)/0.233
Counting	Long	0.394(0.048)/0.042	0.323(0.042)/0.072	0.238(0.027)/0.131
GREMLIN	Long	0.642(0.048)/0.059	0.584(0.045)/0.113	0.455(0.038)/0.215
PSICOV	Long	0.609(0.047)/0.057	0.525(0.042)/0.100	0.360(0.031)/0.167
PhyCMAP	Long	0.414(0.039)/0.039	0.373(0.034)/0.073	0.270(0.023)/0.127
NNcon	Long	0.297(0.031)/0.027	0.263(0.025)/0.050	0.197(0.017)/0.094
DNCON[d]	Long	-	0.326(0.011)/0.052	-
EPC-map	Medium	0.714(0.032)/0.172	0.589(0.031)/0.283	0.405(0.028)/0.455
Counting	Medium	0.558(0.038)/0.136	0.475(0.032)/0.227	0.334(0.026)/0.380
GREMLIN	Medium	0.547(0.045)/0.128	0.450(0.040)/0.212	0.279(0.027)/0.314
PSICOV	Medium	0.501(0.046)/0.111	0.375(0.037)/0.168	0.243(0.025)/0.275
PhyCMAP	Medium	0.490(0.035)/0.110	0.407(0.029)/0.181	0.307(0.022)/0.337
NNcon	Medium	0.354(0.038)/0.078	0.314(0.032)/0.136	0.265(0.023)/0.272
DNCON[d]	Long	-	0.368(0.011)/0.190	-

[a]Accuracy
[b]Standard error
[c]Coverage
[d]Values reported in the original paper of DNCON [9]

Table S16: Accuracies of the single SVM classifiers and the Ensemble SVM on 528 proteins from the CASP9-10_hard, EPC-map_test, D329 and SVMCON_test data sets

Classifier	Range	Acc[a](SE[b])/Cov[c][L/10]	Acc[a](SE[b])/Cov[c][L/5]	Acc[a](SE[b])/Cov[c][L/2]
SVM 1	Long	0.309(0.011)/0.031	0.280(0.009)/0.058	0.218(0.007)/0.113
SVM 2	Long	0.294(0.011)/0.031	0.267(0.009)/0.057	0.220(0.007)/0.114
SVM 3	Long	0.317(0.010)/0.033	0.283(0.009)/0.059	0.222(0.007)/0.115
SVM 4	Long	0.328(0.011)/0.033	0.287(0.009)/0.059	0.220(0.007)/0.135
SVM 5	Long	0.309(0.011)/0.032	0.286(0.009)/0.060	0.224(0.007)/0.117
Ensemble SVM	Long	0.387(0.012)/0.039	0.332(0.010)/0.068	0.255(0.007)/0.131

[a]Accuracy
[b]Standard error
[c]Coverage

References

[1] Cavallo L, Kleinjung J, Fraternali F (2003) POPS: a fast algorithm for solvent accessible surface areas at atomic and residue level. Nucleic Acids Res 31: 3364–3366.

[2] Janda JO, Meier A, Merkl R (2013) CLIPS-4D: a classifier that distinguishes structurally and functionally important residue-positions based on sequence and 3D data. Bioinformatics 29: 3029–3035.

[3] Fischer JD, Mayer CE, Soeding J (2008) Prediction of protein functional residues from sequence by probability density estimation. Bioinformatics 24: 613–620.

[4] Li Y, Fang Y, Fang J (2011) Predicting residue-residue contacts using random forest models. Bioinformatics 27: 3379–3384.

[5] Cheng J, Baldi P (2007) Improved residue contact prediction using support vector machines and a large feature set. BMC Bioinformatics 8: 113.

[6] Frishman D, Argos P (1995) Knowledge-based protein secondary structure assignment. Proteins 23: 566–579.

[7] Li G, Semerci M, Yener B, Zaki MJ (2012) Effective graph classification based on topological and label attributes. Statistical Analysis and Data Mining 5: 265-283.

[8] Hagberg AA, Schult DA, Swart PJ (2008) Exploring network structure, dynamics, and function using networkx. Proceedings of the 7th Python in Science Conference : 11-15.

[9] Eickholt J, Cheng J (2012) Predicting protein residue-residue contacts using deep networks and boosting. Bioinformatics 28: 3066–3072.
